# Functional Trait Variation Among and Within Species and Plant Functional Types in Mountainous Mediterranean Forests

**DOI:** 10.3389/fpls.2020.00212

**Published:** 2020-03-04

**Authors:** Nikolaos M. Fyllas, Chrysanthi Michelaki, Alexandros Galanidis, Eleftherios Evangelou, Joana Zaragoza-Castells, Panayiotis G. Dimitrakopoulos, Christos Tsadilas, Margarita Arianoutsou, Jon Lloyd

**Affiliations:** ^1^Biodiversity Conservation Laboratory, Department of Environment, University of the Aegean, Mytilene, Greece; ^2^Institute of Industrial and Forage Crops, Hellenic Agricultural Organisation “Demeter”, Larisa, Greece; ^3^Geography Department, University of Exeter, Exeter, United Kingdom; ^4^Department of Ecology and Systematics, Faculty of Biology, National and Kapodistrian University of Athens, Athens, Greece; ^5^Department of Life Sciences, Silwood Park, Imperial College London, London, United Kingdom; ^6^School of Marine and Tropical Biology, James Cook University, Cairns, QLD, Australia

**Keywords:** leaf economic spectrum, photosynthesis, respiration, soil properties, climate, elevation gradients, Mediterranean mountains

## Abstract

Plant structural and biochemical traits are frequently used to characterise the life history of plants. Although some common patterns of trait covariation have been identified, recent studies suggest these patterns of covariation may differ with growing location and/or plant functional type (PFT). Mediterranean forest tree/shrub species are often divided into three PFTs based on their leaf habit and form, being classified as either needleleaf evergreen (*Ne*), broadleaf evergreen (*Be*), or broadleaf deciduous (*Bd*). Working across 61 mountainous Mediterranean forest sites of contrasting climate and soil type, we sampled and analysed 626 individuals in order to evaluate differences in key foliage trait covariation as modulated by growing conditions both within and between the *Ne*, *Be*, and *Bd* functional types. We found significant differences between PFTs for most traits. When considered across PFTs and by ignoring intraspecific variation, three independent functional dimensions supporting the Leaf-Height-Seed framework were identified. Some traits illustrated a common scaling relationship across and within PFTs, but others scaled differently when considered across PFTs or even within PFTs. For most traits much of the observed variation was attributable to PFT identity and not to growing location, although for some traits there was a strong environmental component and considerable intraspecific and residual variation. Nevertheless, environmental conditions as related to water availability during the dry season and to a smaller extend to soil nutrient status and soil texture, clearly influenced trait values. When compared across species, about half of the trait-environment relationships were species-specific. Our study highlights the importance of the ecological scale within which trait covariation is considered and suggests that at regional to local scales, common trait-by-trait scaling relationships should be treated with caution. PFT definitions by themselves can potentially be an important predictor variable when inferring one trait from another. These findings have important implications for local scale dynamic vegetation models.

## Introduction

In recent years the study of plant functional traits has gained particular interest in ecological and ecophysiological research. This interest arises from the idea that functional traits can provide a stable basis for re-expressing fundamental ecological processes from first principles (McGill et al., 2006): this even leading to the suggestion that there may be some sort of ecological equivalence to the elemental periodic table (Winemiller et al., 2015). Recently developed global plant traits databases (Kattge et al., 2011), and analyses of trait co-variation across wide geographical scales (Reich et al., 1997; Díaz et al., 2016), have provided valuable inroads toward that objective. Nevertheless, considerable intra-specific variation at local scales and environmentally induced variation at larger scales have been repeatedly observed, with their implications still not fully explored (Siefert et al., 2015).

Comparative studies of functional trait variation across species, or plant functional types (PFTs), provide one basis for the identification of life history strategies (Reich et al., 2003; Adler et al., 2014) and parameterisation of dynamic vegetation models (Fyllas et al., 2012; Atkin et al., 2015). Some common leaf, wood, and seed traits are assumed to reveal the way plants acquire resources, reproduce, and compete with other plants (Westoby et al., 2002). For example, leaf area (*L*_a_) variations reflect some aspects of the whole plant leaf energy and water balances; leaf dry mass per area (*LMA*) and leaf nutrients concentration variations may reflect contrasting resource allocation strategies; seed mass (*S*_m_) variations reflect seedling survival and colonisation trade-offs; maximum plant height (*H*_max_) variations are indicative of a plant’s ability to capture light and disperse seeds; and wood density (ρ_W_) variations broadly reflect a trade-off between growth and mortality (Díaz et al., 2016). Although these general dimensions of trait variation that identify fundamental plant strategies have been observed globally, recent studies suggest that at local scales these relationships may not be robust (Messier et al., 2017) and with trait-by-trait scaling relationships differing between sites characterised by different species and/or growing conditions (Schrodt et al., 2015; Lira-Martins et al., 2019) or within species (Anderegg et al., 2018). This means that generic equations that predict one trait from another may not be possible across a broad spectrum of scales. Functional trait variation can be related to species taxonomy as well as to the environmental conditions a particular individual is growing under (Fyllas et al., 2009; Garnier et al., 2016). Geographic gradients, where a set of species is repeatedly found under different conditions, provide natural laboratories for exploring the relative effects of taxonomy and environmental plasticity on trait variation (Asner et al., 2014; Turnbull et al., 2016).

Mountainous Mediterranean forests (MMF) occur across extended mountain ranges, such as those of the Grecian Peninsula, where forest type frequently covaries with elevation from relatively dry Mediterranean to more temperate profiles (Box and Fujiwara, 2015). Three basic woody PFTs dominate these mountains: needleleaf evergreen (*Ne*), broadleaf deciduous (*Bd*) and sclerophyllous broadleaf evergreen (*Be*). These PFTs are usually *a priori* defined based on their leaf form and habit with the *Ne* and *Be* species typically having higher leaf longevity than *Bd*. Nevertheless an evaluation of other functional traits is necessary to better understand and model the underlying basis of their differential distributions across the Mediterranean region (Carnicer et al., 2013), or their contribution across communities of different successional stage and/or disturbance history (José Vidal-Macua et al., 2017). Understanding how these PFTs interact with each other and why a particular PFT may dominate under particular environmental conditions is important in order to predict vegetation dynamics under global change at both the regional and planetary scale.

Needleleaf evergreen species are mainly found in relatively adverse environments and disturbed habitats and are considered to be able to survive under extreme conditions due to their relatively high cavitation resistance and nutrient use efficiency (Bond, 1989; Brodribb et al., 2012). The high cavitation resistance of *Ne* species is related to the wide hydraulic safety margin under which they operate (Choat et al., 2012) and thus a generally lower hydraulic conductivity, which may, in turn, reduce their competitive ability against angiosperms under favourable conditions (Reich et al., 2003). However, recent studies suggest that single hydraulic traits should not be used to explain differences in whole plant hydraulic strategy (Anderegg, 2015; Gleason et al., 2016), that PFT classification might not adequately capture the impacts of drought on tree mortality (Anderegg, 2015) and that the severity of drought might be a stronger predictor of tree mortality compared to PFT grouping (Greenwood et al., 2017). *Ne* species tend to be superior colonisers in disturbed sites but they are also able to tolerate low disturbance regimes (Brodribb et al., 2012). Under conditions of high and stable water and nutrient availabilities, broadleaf deciduous species are considered to generally outcompete *Ne* (Berendse and Scheffer, 2009). This is thought to be due to their higher hydraulic conductivity, lower *LMA*, and higher photosynthetic capacity which place them toward the ‘acquisitive’ part of the leaf economic spectrum (Wright et al., 2004). Broadleaf evergreen species of MMF on the other hand, prevail at drier conditions (Box and Fujiwara, 2015) by deploying long-lived schlerophyllous leaves, with high construction costs (Williams et al., 1989) and high *LMA* that can survive extended dry periods and maintain photosynthesis through and beyond periods of extended soil water deficits (Givnish, 2002; De Micco and Aronne, 2012).

Recent ecophysiological studies suggest that the contrasting trait syndromes between angiosperms and gymnosperms, can lead to different responses to increased temperature and drought (Carnicer et al., 2013). It is thus important to understand how functional traits express ‘plant strategies,’ and if trait covariation differentiates between PFTs. In this study we therefore systematically measured 12 leaf morphological and biochemical traits plus wood density across a range of species and environmental conditions all along a forest plot network on Mediterranean mountains in Greece (Table 1). In addition, we estimated species-specific maximum height from tree-by-tree biometric measurements made within each plot, and we extended the trait database with seed mass information from the literature. We aimed to: (1) test whether PFT definition in MMF correspond to different suites of functional traits, (2) test if the patterns of trait covariation are similar within PFTs, and (3) interpret the effects of environmental variation on functional trait variation at the PFT and the species level.

**TABLE 1:** Functional traits and environmental variables abbreviations and units of measurement used in this study.

**Functional trait**	**Abbreviation**	**Unit**
Leaf area	*L*_a_	cm^2^
Leaf dry mass per area	*LMA*	g m^–2^
Leaf dry matter content	*LDMC*	g g^–1^
Leaf thickness	*L*_t_	mm
Leaf C concentration	*C*_m_	mg g^–1^
Leaf N concentration	*N*_m_	mg g^–1^
Leaf P concentration	*P*_m_	mg g^–1^
Leaf Ca concentration	*Ca*_m_	mg g^–1^
Leaf Mg concentration	*Mg*_m_	mg g^–1^
Leaf K concentration	*K*_m_	mg g^–1^
Light saturated photosynthetic rate on area basis	*A*_sat,a_	mmol m^–2^ s^–1^
Dark respiration rate on area basis	*R*_dark,a_	mmol m^–2^ s^–1^
Wood density	ρ_W_	g cm^–3^
Seed mass	*S*_m_	g
Maximum tree height	*H*_max_	m
**Environmental variable**		
Average annual temperature	*T*_A_	°C
Average monthly temperature	*T*_i_	°C
Minimum temperature of the coldest month	*T*_min_	°C
Annual precipitation	*P*_A_	mm
Monthly precipitation	*P*_i_	mm
Driest quarter precipitation	*P*_dq_	–
Soil sand content	Sand	%
Soil clay content	Clay	%
Soil pH	pH	
Soil electric conductivity	EC	μs cm^–1^
Soil organic matter	SOM	%
Soil N concentration	N	%
Soil P concentration	P	%
Soil K concentration	K	cmol kg^–1^
Soil Ca concentration	Ca	cmol kg^–1^
Soil Mg concentration	Mg	cmol kg^–1^
Water holding capacity	WHC	%

## Materials and Methods

### Study Plots and Species

The study extended across 61 plots of the MEDIT network (Fyllas et al., 2017b) and covered the most important mountains of continental Greece in terms of both species diversity and ecosystem productivity (Supplementary Figure S1). Sites sampled covered an altitudinal range from 374 m above sea level (asl) at Mount Olympos to 1665 m asl at Mount Parnassos, with mean annual temperatures (*T*_A_) varying from 5.9 (1516 m asl at Mount Rodopi) to 15.0°C (470 m asl at Mount Parnitha), and with mean annual precipitations (*P*_A_) ranging from 0.37 (470 m asl at Mount Parnitha) to 1.1 m (1358 m asl at Mount Pindos) (Supplementary Table S1). On each of the 15 mountains, we established at least three 30 m × 30 m plots at different elevations: ‘low,’ ‘medium,’ and ‘high.’ In some mountains were many forest and soil types were abundant we established more than three plots, for example at Mounts Rodopi, Pindos, Olympos, and Kissavos. In each plot, diameters at breast (1.3 m) height (*D*) were determined for all individuals with *D* ≥ 10 mm. We also estimated the height (*H*) of more than 50% of the individuals in each plot, ensuring to cover the full range of observed *D*. Five soil pits to 0.3 m depth were also dug in each plot and all soil, below the litter horizon, was extracted for subsequent analysis of physical and chemical properties. Plot-level leaf area index (*LAI*) was estimated using an ACCUPAR 2000 as the average values of 20 measurements made at 1 m above the forest floor.

Within our plots we sampled individuals from 39 tree and shrub species. Individuals from the 24 dominant species (defined as those species that contributed at least 5% to the total stand basal area) were used for further analysis. Each species was assigned to one of the three PFTs already discussed, *viz* needleleaf evergreens including *Abies cephalonica* Loudon, *Abies borisii-regis* Mattf., *Picea abies* Karst., *Pinus halepensis* Miller, *Pinus nigra* Arnott and *Pinus sylvestris* L., broadleaf evergreens including *Arbutus unedo* L., *Arbutus andrachnae* L., *Quercus coccifera* L., *Quercus ilex* L., and *Phillyrea latifolia* L. and broadleaf deciduous including *Acer campestre* L., *Betula pendula* Roth, *Carpinus orientalis* Mill*., Castanea sativa* Mill., *Corylus avellana* L., *Cotynus coggygria* Scop., *Fagus sylvatica* L., *Fraxinus ornus* L., *Ostrya carpinifolia* Scop*., Pistacia terebinthus* L., *Quercus cerris* L., *Quercus frainetto* Ten., and *Quercus pubescens* Willd.

### Functional Trait Measurements

Within each plot (apart from the monospecific stands) at least 10 individuals were selected for functional trait measurements (Supplementary Table S12). The number of individuals per species was selected based on the relative contribution of each species to the stand’s basal area. One, fully sunlit branch from mature individuals was cut by climbing on the tree and/or using telescopic scissors, and immediately placed in a water bucket where it was recut prior to leaf gas-exchange measurements. For species found only in the understory, sunlit individuals outside the plot were sampled. Whilst still attached to the recut branch, healthy fully expanded leaves were selected and placed within the gasket of a LICOR-6400 infrared gas analyser (LI-COR, Lincoln, NE, United States). Only current year’s leaves were used. Gas-exchange was monitored and when leaves had reached a stable photosynthetic rate, with a stomatal conductance higher than 0.05 μmol s^–1^ m^–2^ at an incident photon irradiance (*I*) of 1500 μmol quanta m^–2^ s^–1^, the area-based light saturated net photosynthetic rate (*A*_sat,a_ in μmol CO_2_ s^–1^ m^–2^) was recorded as the mean value of five measurements per leaf, made across 3 s intervals. The area-based leaf respiration rate (*R*_dark,a_ in μmol CO_2_ s^–1^ m^–2^) was estimated from the average value of five (with 3 s intervals) measurements made on leaves that were placed for at least 5 min in the dark. All measurements were made with a chamber temperature near 25°C and a relative humidity between 50 and 70%. The average temperature of the chamber during the measurements was recorded, and subsequently both *A*_sat,a_ and *R*_dark,a_ were re-expressed at a common temperature of 25°C, using the equations from Tjoelker et al. (2001) and Higgins et al. (2016) respectively.

In addition to the leaves used in the *A*_sat,a_ and *R*_dark,a_ measurements, at least two, fully developed leaves of the same branch were placed in sealed bags with moist tissue paper and left in dark conditions for 24 h before their water-saturated leaf fresh mass (*W*_s_ in g) was measured. Laminar leaf thickness (*L*_t_ in mm) was measured with a digital calliper, the leaves were scanned with a portable scanner and the projected leaf area (*L*_a_ in cm^2^) was estimated using the image analysis software *Image-J* (NHI, version 1.47). Once back in the laboratory, leaves were dried at 80°C for 48 h and their dry weight (*W*_d_ in g) determined. The leaf dry matter content (*LDMC* in g g^–1^) was subsequently determined as the ratio of *W*_s_/*W*_d_. Leaf dry mass per unit area (*LMA* in g m^–2^) was estimated as the ratio of *W*_d_ to *L*_a_. The mass-based photosynthesis (*A*_sat,m_) and dark respiration (*R*_dark,m_) rates were calculated by dividing the area-based rates with *LMA*. For the determination of wood density (ρ_W_ in g cm^–3^), a piece of each cut branch was transferred to the lab, where its dry weight (at 70°C for 48 h) and volume, estimated via the water displacement method, were measured (Williamson and Wiemann, 2010).

For determinations of leaf cations and P composition, 0.5 g of ground leaf material was heated at 450°C for 5 h, in 1N HCl and with Ca and Mg concentrations subsequently determined using an atomic absorption spectrophotometer, K with a Corning 410 flame photometer, and P by the vanade-molybdate method (Jones et al., 1991). Plant N content (1 g samples) was determined by the Kjeldahl wet-oxidation method (Bremner and Mulvaney, 1982). Total C and S were determined by a LECO CNS 2000 analyser (TruSpec Micro, St. Joseph, MI, United States). All leaf nutrient concentrations are expressed on a per mass basis (in mg g^–1^, denoted hereafter with a ‘m’ subscript, for example *N*_m_) with N and P additionally expressed and used in statistical analyses on an area basis (*N*_a_, *P*_a_) due to the obvious relevance of area based metrics when looking at photosynthesis-nutrient associations (Lloyd et al., 2013).

For some analyses, the functional trait dataset of leaf and wood traits, was complemented by estimates of maximum species height and seed mass. Maximum species height (*H*_max_ in m) was approximated using the 0.99 quantile from the tree-by-tree measurements across the plot network (Supplementary Table S2). Seed mass (*S*_m_ in g) data for all species were extracted from the *Seed Information Database* (Royal Botanic Gardens Kew, 2018).

### Climatic Data and Edaphic Properties

For each plot, long-term high resolution (∼1 km^2^) climate data were extracted from the CHELSA database (Karger et al., 2017), including average monthly (*T*_i_) and annual temperature (*T*_A_), total monthly (*P*_i_) and annual precipitation (*P*_A_), and total precipitation during the driest quarter of the year (*P*_dq_).

In the lab, composite soil samples (from the five pits) were air dried, crushed and 2 mm sieved, prior to determination of soil particle size by the hydrometer method (Bouyoucos, 1962). Soil pH and electrical conductivity were also estimated in a suspension of 1:1 water:soil (Doran et al., 1996), organic C determined by the Walkley–Black wet oxidation method (Nelson and Sommers, 1982), and total N by Kjeldahl wet-oxidation (Bremner and Mulvaney, 1982). Soil total P was determined by wet-acid digestion with HNO_3_ and H_2_O_2_ (Jones et al., 1991).

The exchangeable cations K, Ca, and Mg were extracted with 1N ammonium acetate at pH 7 (Thomas, 1982), with K concentrations subsequently measured by Corning 410 flame photometer, and Ca and Mg, by Varian AA400 Plus atomic absorption. Carbonate content was determined using the Bernard method by measuring the evolved CO_2_ after addition of HCl (Nelson, 1982). Maximum water holding capacity (WHC) was measured for each soil sample (Gardner, 1986): each soil sample was saturated with water in a cylinder, and WHC was calculated based on the weight of the water held in the sample vs. the sample dry mass (dried at 105°C for 24 h). By measuring WHC without taking into account stone and/or rock content, we refer to the intrinsic ability of mineral soils to hold water regulated mainly by pedogenetic factors that determines soil type and soil organic matter dynamics.

### Statistical Analysis

All statistical analyses and figures were made with R (R Core Team, 2019). Initially, a linear discriminant analysis (LDA, package *MASS*) was performed on the full trait dataset (including intraspecific variation) to verify that functional traits (predictor variables) can be efficiently used to define PFTs (response variable). Analyses of variance (log_10_ transformed data, apart from ρ_W_), followed by Tukey HSD *post hoc* tests were used to explore for differences among mean trait values between the three PFTs, with species treated as a random effect (packages *lme4*, *lsmeans*) and including intraspecific trait variation by using the full dataset. Within angiosperms we additionally applied a phylogenetic ANOVA (package *phytools*; Revell, 2012) to test for trait differences between *Bd* and *Be* species, using in this case the across site mean trait values per species. The latest GBOTB tree was used to take into account the phylogenetic history of the study species (package *V.PhyloMaker*; Jin and Qian, 2019). The correlation matrix of the per species average trait dataset (no intraspecific variation), extended with the species-specific mean *H*_max_ and *S*_m_, was analysed with a Principal Components Analysis (PCA, package *FactoMineR*), to identify the major functional dimensions. Further PCAs were performed, at the full dataset (including intraspecific variation) as well as after aggregating the full dataset into PFTs (*Ne*: *n* = 128, *Bd*: *n* = 181, *Be*: *n* = 35), so as to explore whether the major functional dimensions differed between PFTs. In these analyses *H*_max_ and *S*_m_ were excluded as data was not available for each measured individual. For all PCAs we estimated the radius of the equilibrium circle of descriptors (Legendre and Legendre, 1998) to assess the contribution of each trait to each principal component.

For both the full-dataset, as well as testing separately within each PFT, Pearson’s correlation coefficients were estimated for all trait pairs. Analyses were performed on log_10_ transformed traits values, with the exception of ρ_W_. For significantly correlated trait pairs, standardised major axis regressions (SMA, package *smart*) were fitted in order to test whether the scaling relationships between traits were similar across PFTs.

Each trait’s variability was estimated by the coefficient of variation (CV). In addition multilevel linear models (package *lme4*) were used to quantify the sources of trait variation (Fyllas et al., 2009) that account for: (a) between PFT variation (Φ), (b) interspecific variation (*S*), (c) variation among regions (*R*), i.e., between mountains (this reflecting plastic and/or filtering responses to the wide environmental gradient (climatic and edaphic) of our plot network), and (d) between plot variation (*P*), this reflecting natural environmental variability of plant growing conditions. Our multilevel model can be written as:

(1) T=μ+Φ/S+R/P+ε

with μ, the overall mean value for each trait (*T*), and ε the residual term, which includes both within-species variability, as well as any measurement error. As only sun-leaves were collected, we expect micro-environmental effects to be minor in comparison to taxonomic and environmental effects.

After fitting the multilevel model for each trait, the derived components were extracted for further analysis. In particular the environmental effect on each trait’s variation was estimated by adding the regional and plot (*E* = *R* + *P*) components. The *E* component expresses the value a trait would take in each plot after removing the effects of PFTs and species, revealing the ‘true’ effect of environmental variability (Fyllas et al., 2009). Climatic variability between plots was expressed by variation in the minimum temperature of the coldest month (*T*_min_) and the total precipitation of the driest quarter (*P*_dq_). These two variables were used to express the two main climate factors limiting plant growth in Mediterranean plants during the year, i.e., winter cold and summer drought (Mitrakos, 1980). In order to identify the key axes of edaphic variability across our plot network a PCA on the correlation matrix of the soil variables was performed, and the scores of the plots on the first two axes were used as edaphic predictors for subsequent analyses. We note that our soil measurements were made at the top 30 cm and might not be appropriate for species with deep roots, but can in general be considered proxies for soil fertility and water retention ability (Ordoñez et al., 2009). The effects of local growing conditions (climatic and edaphic) on each trait’s environmental component were tested using Kendall partial correlation analysis (package *ppcorr*).

Finally, for a subset of four species (*Abies cephalonica, Pinus nigra, Fagus sylvatica*, and *Quercus frainetto*), with functional trait measurements in at least five individuals of these species across a minimum of five plots, linear mixed effect models were used to explore whether the effects of environmental variability on trait variation were independent of species identity. Trait values were *z*-score standardised, based on the trait mean value and standard deviation, making effect sizes comparable across traits. In this analysis, *LAI* (index of light availability) and the two climatic and two edaphic gradients were used as fixed effects, with species and plot used as random effects. For model selection and validation we started with models that used all fixed effect terms, and searched for the optimal random structure, by systematically allowing both intercepts and slopes to vary for each species across the climatic and edaphic gradients of the plot network (Zuur et al., 2009). All models were fitted using REML and the model with the lowest AIC was selected. Subsequently we searched for the optimal fixed components by sequentially removing the non-significant fixed-effect terms (package *lmerTest*).

## Results

### Plant Functional Types in MMF

**FIGURE 1:**
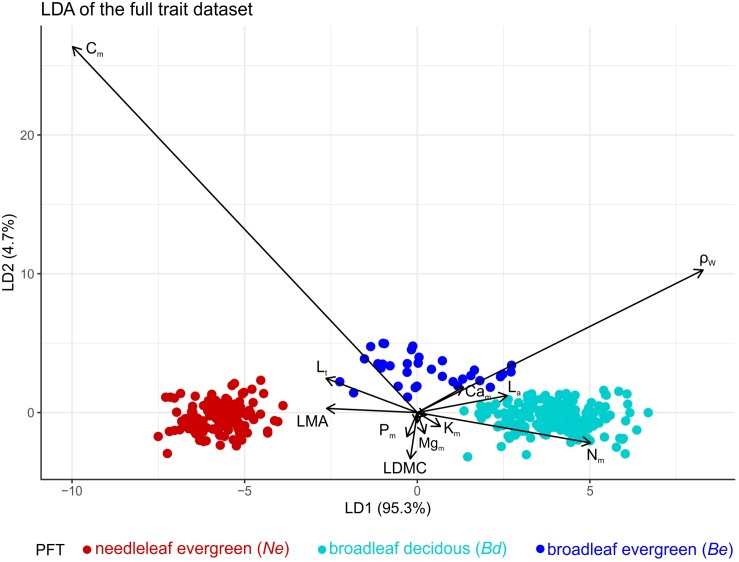
Linear discriminant analysis on the full trait dataset indicating an effective separation of PFTs based on their 10 leaf and one wood trait values. Colours indicate different plant functional types (PFTs) (*Ne*: needleleaf evergreens, *Be*: broadleaf evergreens, and *Bd* broadleaf deciduous). Trait abbreviations: *L*_a_, leaf area; *LMA*, leaf dry mass per area; *LDMC*, leaf dry matter content; *L*_t_, leaf thickness; *N*_m_ – *P*_m_ – *Ca*_m_ – *Mg*_m_ – *K*_m_ leaf, N, P, Ca, Mg, and K mass basis concentrations, *A*_sat,a_, light saturated photosynthetic rate on area basis; *R*_dark,a_, dark respiration rate on area basis and ρ_w_ wood density. See Table 1 for units.

Linear discriminant analysis found the first axis (LD1) to explain 95% of the total variance, with LD1 being negatively correlated with leaf *C*_m_ content and ρ_W_ and positively with *N*_m_. Needleleaf evergreen and broadleaf deciduous species were perfectly separated with a small overlap between evergreen and deciduous broadleaves (Figure 1). This analysis provided good support for our *a priori* PFT definition.

**FIGURE 2:**
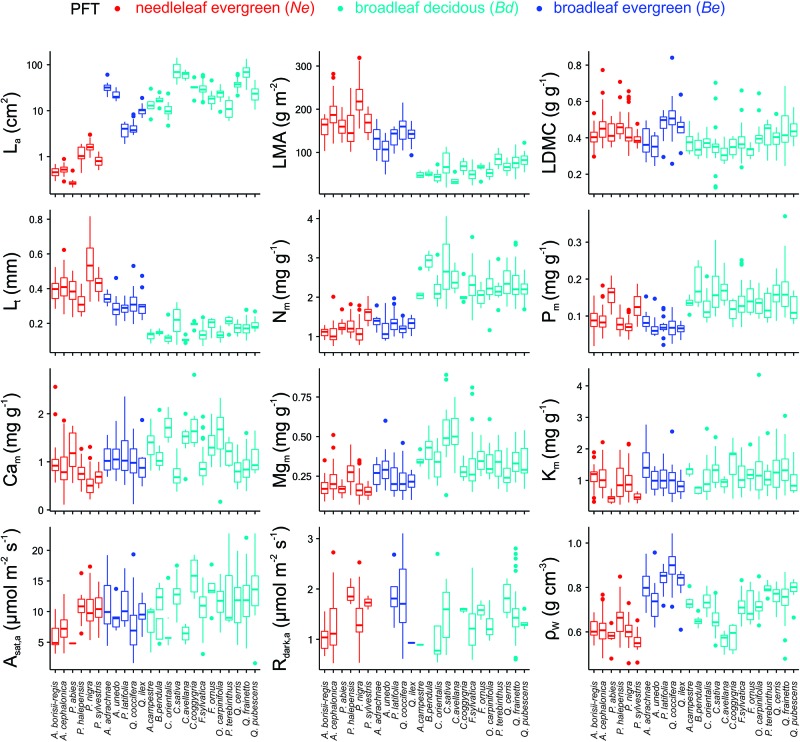
Mean trait values for the 24 dominant species of the MEDIT plot network (see also Supplementary Table S3). See Table 1 for abbreviations and units.

Differences both within and between PFT for most of the studied traits were identified (Figure 2 and Supplementary Tables S3, S4). For example, *Ne* had lower mean *L*_a_, ρ_W_ and *A*_sat,m_ than either of the broadleaf PFTs, *Bd* had the highest *N*_m_, *P*_m_, and *Mg*_m_ concentrations and the highest *A*_sat,m_ and *R*_dark,m_ and *Be* had the highest ρ_W_. The differences between the two broadleaf PFTs were maintained even when their phylogenetic history was considered (Supplementary Table S5), with *Be* having a lower *N*_m_, *P*_m_, *Mg*_m_, *A*_sat,a_, and *R*_dark,a_ but higher *LMA*, *L*_t_, *C*_m_ and ρ_W_ than *Bd.* Although there was no difference between the three PFTs in terms of their mean *A*_sat,a_, when comparing within PFT there were substantial differences between species evident (for example *A. borisii-regis* vs. *P. halepensis* and *C. avellana* vs. *C. coggygria*).

**FIGURE 3:**
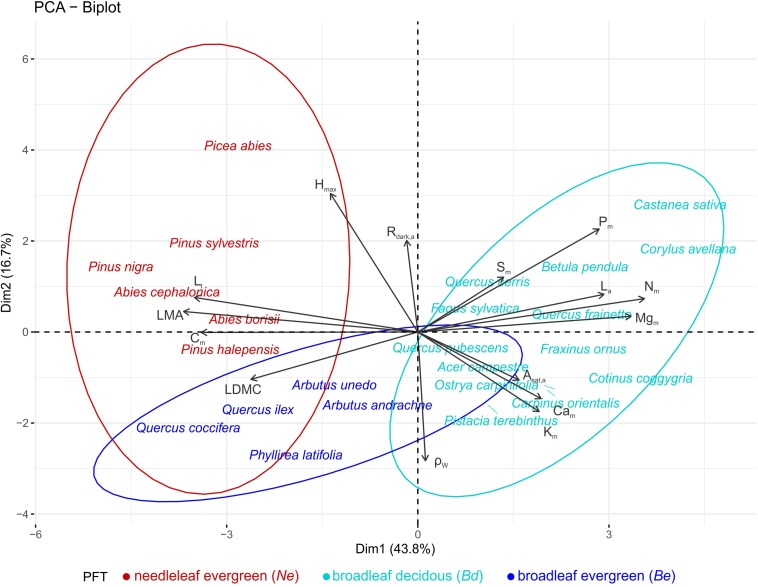
Principal components analyses (first two axes) on the average species traits dataset (15 traits), for the 24 most dominant species of the MEDIT plot network. Colors indicate different PFTs (*Ne*: needleleaf evergreens, *Be*: broadleaf evergreens, and *Bd*: broadleaf deciduous). See Table 1 for abbreviations and units.

A PCA on the species means dataset, identified three functional dimensions (Table 2). The first dimension (PC1 – 44% of total trait variance) can be considered to describe a leaf dimension that contrasts thick leaves with high *LMA*, *LDMC*, and *C*_m_ with large nutrient rich (N, P, Mg) leaves: separating species across a leaf construction cost dimension. The second dimension (PC2 – 17%) is positively related with *H*_max_ and *R*_dark,a_, and negatively with ρ_W_, and could be considered to reflect a trade-off between height gain and persistent life strategy. The third axis (12% of total variance) was mainly related to seed mass. The 24 studied species occupied distinct areas of the multidimensional trait space (Figure 3): *Ne* occupying the high leaf construction cost (high PC1 scores), *Bd* having low construction cost/high nutrient (dry weight basis) leaves (low PC1 scores), while *Be* seem to adopt an overall conservative tissue construction strategy (low PC1) coupled with small adult stature and high ρ_W_ (low PC2 scores).

**TABLE 2:** Principal components analysis on 15 traits expressing whole-plant economics, aggregated at species level, for the 24 most dominant species.

	**PC1**	**PC2**	**PC3**
Eigenvalue	6.56	2.51	1.87
Portion of variance	43.74	16.76	12.46
*L*_a_	**0.76**	0.21	0.46
*LMA*	**−0.95**	0.12	0.16
*LDMC*	**−0.91**	0.20	0.12
*L*_t_	**−0.68**	−0.27	0.33
*C*_m_	**−0.88**	0.00	0.25
*N*_m_	**0.92**	0.19	0.06
*P*_m_	**0.74**	0.59	−0.07
*Ca*_m_	0.50	−0.37	**−0.67**
*Mg*_m_	**0.87**	0.08	0.10
*K*_m_	0.49	−0.45	0.33
*A*_sat,a_	0.41	−0.28	0.40
*R*_dark,a_	−0.05	**0.53**	0.02
ρ_W_	0.03	**−0.73**	0.38
*S*_m_	0.35	0.31	**0.76**
*H*_max_	−0.34	**0.80**	0.01

*Variables that contribute more than the radius of the equilibrium contribution circle (*r* = 0.45), at each principal component (PC), are indicated with bold. Trait abbreviations: *L*_*a*_, leaf area; *LMA*, leaf dry mass per area; *LDMC*, leaf dry matter content; *L*_*t*_, leaf thickness; *N*_*m*_ – *P*_*m*_ – *Ca*_*m*_ – *Mg*_*m*_ – *K*_*m*_, leaf N, P, Ca, Mg, and K mass basis concentrations; *A*_*sat,a*_, light saturated photosynthetic rate on area basis; *R*_*dark,a*_, dark respiration rate on area basis; ρ_*w*_, wood density; *S*_*m*_, seed mass; *H*_*max*_, maximum tree height.*

**FIGURE 4:**
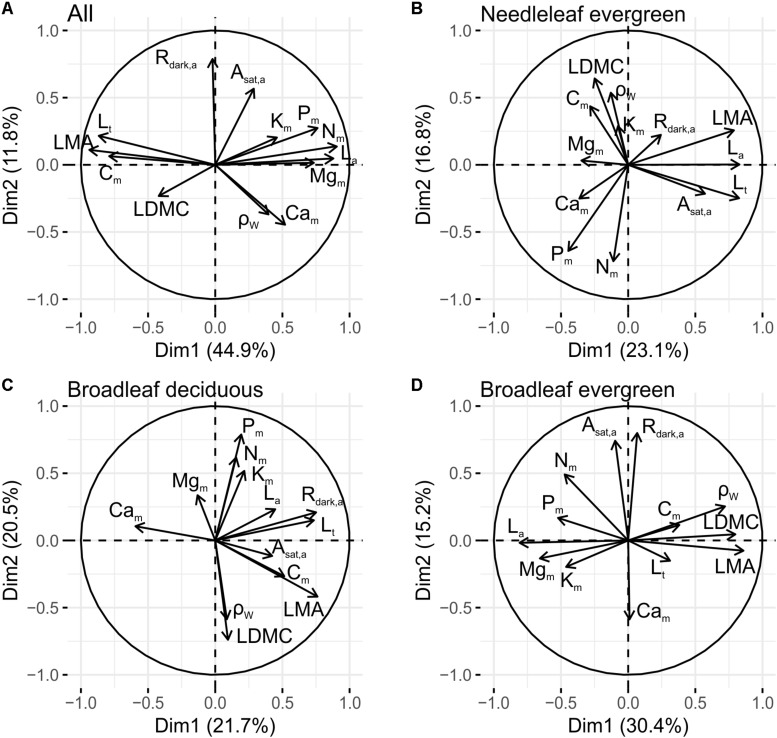
Principal components analyses of 12 foliar and 1 wood traits, across PFTs **(A)** within needleleaf evergreens **(B)**, within broadleaf deciduous **(C)**, and within broadleaf evergreens **(D)** (see also Supplementary Table S6). See Table 1 for abbreviations and units.

Further probing for PFTs differences through an assessment of the trait inter-relationships within each PFT (i.e., treating each tree as an separate observation rather than using species means as above), the first two axes of the full dataset PCA explained 64% of the total variance (Supplementary Table S6). PC1 (45%) was strongly positively related to *L*_a_, *N*_m_, *P*_m_, and *Mg*_m_ and negatively to *LMA*, *L*_t_, and *C*_m_ (Figure 4A). This is similar to the first dimension identified in the species level analysis above. On the other hand PC2 (12%) was in this case mainly related to *A*_sat,a_ and *R*_dark,a_, suggesting that leaf gas exchange, expressed on an area basis, is largely independent to leaf resource allocation. PC3 (10%) was associated to *LDMC* and ρ_W_, representing a tissue toughness dimension.

Considering then each PFT separately, for *Ne* the first PC (23%), represented a needle size dimension, with *L*_a_ covarying with *LMA*, *L*_t_, and *A*_sat,a_ (Figure 4B). The second conifer axis (17%) was associated positively to *LDMC* and ρ_W_ and negatively to *N*_m_ and *P*_m_. Thus in *Ne* species leaf construction and gas exchange seems to be independent from *N* and *P* concentration, at least when the nutrients are expressed on a dry-weight basis. On the other hand, tissue density (expressed by *LDMC* and ρ_W_) are both negatively associated with *N*_m_ and *P*_m_.

For *Bd*, the first PC (22%) was positively related to *LMA*, *L*_t_ and *R*_dark,a_, indicating that thicker leaves have a higher per area maintenance cost (Figure 4C) and similar to *Ne*, the second *Bd* dimension (21%) contrasts leaves of high nutrient investment (mainly *N*_m_, *P*_m_, and *K*_m_) with leaves of high *LDMC* and ρ_W_.

Although the PCA results for the *Ne* and *Bd* were broadly similar, for *Be* a different pattern was observed with the first dimension (30%) positively related to *LMA*, *LDMC*, and ρ_W_ and negatively to *L*_a_ and *Mg*_m_. This indicates that within this PFT leaf and wood construction traits integrate along a common axis (Figure 4D). The second axis (15%) is positively related to *A*_sat,a_ and *R*_dark,a_ and negatively to *Ca*_m_, suggesting for this PFT that variations in photosynthetic capacity and respiration are independent of leaf construction costs.

### Differences in Bivariate Trait Relationships Between PFTs

**FIGURE 5:**
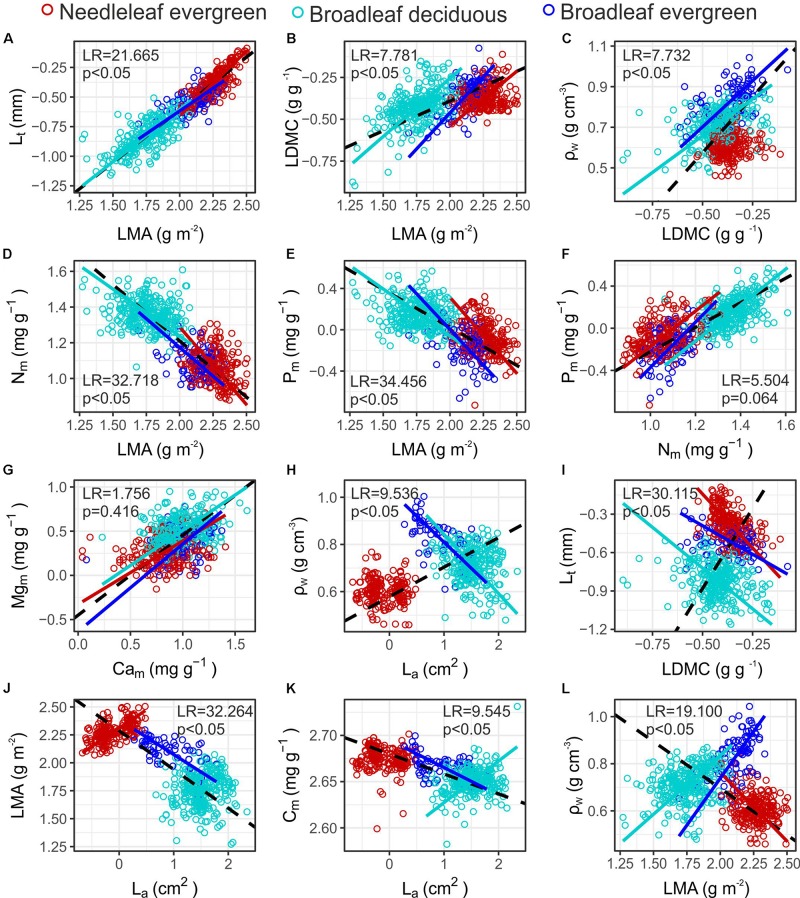
Bivariate relationships among functional traits in Mediterranean Forests **(A–L)**. Colours indicate individuals’ PFT. When a significant relationship was identified a SMA fit is shown in the respective colour, broken black lines indicate significant relationships in the full dataset (see Supplementary Table S7 for coefficient estimates). The LR tests indicate significant differences between the slope of the PFT specific SMA lines. See Table 1 for abbreviations and units.

Numerous significant bivariate trait relationships were identified both within and across PFTs (Supplementary Figure S2 and Supplementary Table S7). In many cases the sign of the association was similar between PFTs, for example the positive relationships between *L*_t_ – *LMA* (Figure 5A), *LDMC* – *LMA* (Figure 5B) and *LDMC* – ρ_W_ (Figure 5C). Similarly, leaf *N*_m_ and *P*_m_ concentration scaled negatively with *LMA* for all groups (Figures 5D,E). Only in few cases a common slope relationship was identified, for example between leaf *N*_m_ and *P*_m_ and between *Mg*_m_ and *Ca*_m_ (Figures 5F,G). However, in most cases (78%) the common slope test indicated a significant difference in the scaling exponent between PFTs, suggesting that scaling relationships depend on PFT for most of the bivariate trait associations examined. Interestingly, when data across PFTs were pooled, some of the relationships become of an opposite sign than when the different PFTs are considered separately. For example, although a significant positive association was identified between ρ_W_ – *L*_a_ (Figure 5H) and *L*_t_ – *LDMC* (Figure 5I) in the full dataset, negative relationships were revealed when considered within PFTs. Furthermore sign-differences emerged even between PFTs. For example *L*_a_ – *LMA* scaled positively within *Ne*, showed no association within the *Bd*, and had a negative association within *Be* (Figure 5J). *L*_a_ – *C*_m_ scaled positively within *Bd* and negatively within *Be* species (Figure 5K). *LMA* scaled negatively with ρ_W_ within *Ne*, and positively within *Be* and *Bd* (Figure 5L).

**FIGURE 6:**
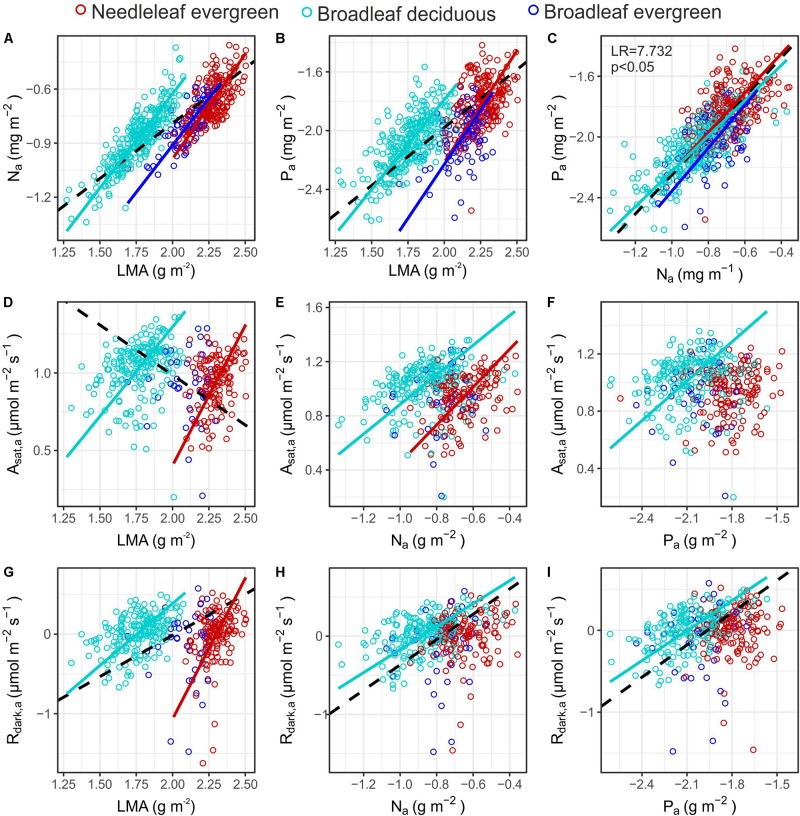
Bivariate relationships between gas exchange rates (*A*_sat,a_ and *R*_dark,a_) and leaf dry mass per area (*LMA*), nitrogen (*N*_a_) and phosphorus (*P*_a_) area content **(A–I)**. Colours indicate individuals’ PFT. When a significant relationship was identified a SMA fit is shown in the respective colour, broken black lines indicate significant relationships in the full dataset (see Supplementary Table S7 for coefficient estimates). The LR tests indicate significant differences between the slope of the PFT specific SMA lines. See Table 1 for abbreviations and units.

Although all three PFTs showed similar positive slopes in their *N*_a_ – *LMA* and *P*_a_ – *LMA* relationships (Figures 6A,B), at any given *LMA*, both *N*_a_ and *P*_a_ were higher for *Bd* than either *Ne* or *Be* (Supplementary Table S7). A contrast between *Ne* and *Bd* was also evident for the relationships between *A*_sat,a_ and both *LMA* and *N*_a_ where the bivariate associations differed in terms of slope and intercept respectively (Figures 6D,E), although in both cases no significant relationship was observed for *Be*. This was also the case for the *R*_dark_ – *LMA* association (Figure 6G). Also of note in Figure 6 is that, despite significant associations being observed for the *Bd A*_sat,a_ – *P*_a_, *R*_dark,a_ – *N*_a_ and *R*_dark,a_ – *P*_a_ associations, no significant relationships were found for these three bivariate associations for either *Ne* or *Be*. Also, despite most of the area-based bivariate associations of Figure 6 being PFT dependent, for the *P*_a_ – *N*_a_ association all three PFTs essentially fall along the same line. Finally we note that, as for some of the relationships in Figure 6, when the data are pooled (without consideration of PFT) the slope of the *A*_sat,a_ vs. *LMA* relationship (Figure 6D) appears negative, even though for both the *Ne* and *Bd* groupings the within PFT-association is clearly positive.

### Trait Variation as Influenced by Plot Location, Plant Functional Type, and Species

**FIGURE 7:**
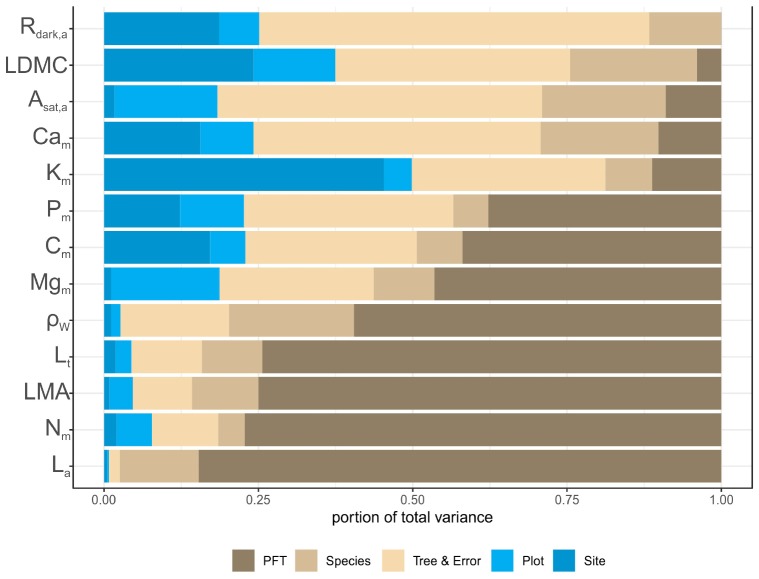
Partitioning of the total variance for leaf and wood functional traits. Traits are sorted based on the portion of variance attributed to PFT. See Table 1 for abbreviations and units.

Of the 13 studied traits the least variable was leaf *C*_m_ content (CV = 0.039) while the most variable was *L*_a_ (CV = 1.219) followed by *LMA*, *L*_t_ and *R*_dark,a_ (Supplementary Table S8). Partitioning this variation according to Eq. 1, for most traits the proportion of the variance attributable to PFT (Φ) and species (*S*) components, surpassed that attributed to environmental conditions (plot and region effects) (Figure 7). For example, for *L*_a_ (0.85), *N*_m_ (0.77), *LMA* (0.75), *L*_t_ (0.74) and ρ_W_ (0.60), most of the variation was attributed to the PFT grouping. By contrast, the environmental component was greater for *K*_m_ (0.50), *L*_dmc_ (0.37) and *R*_dark,a_ (0.25), suggesting that these traits are also considerably influenced by sampling location. For *C*_m_ (0.42 + 0.07), *P*_m_ (0.38 + 0.06), *Mg*_m_ (0.46 + 0.10) and *A*_sat,a_ (0.09 + 0.20), the Φ + *S* component was higher than the environmental component, while the variation of *Ca*_m_ was equally attributable between environment (0.24) and taxonomy (0.29). We note that for most traits there was a significant within species variation and error term, particularly high for *Ca*_m_, *A*_sat,a_, and *R*_dark,a_.

### Environmental Effects

Two main axes of edaphic variation across our plot network were identified. The first PCA axis (48%) was positively correlated with WHC, pH, SOM, N, P, and Ca concentrations, thus generally reflecting ‘Soil Nutrient Status’ variations, while the second was mainly related to ‘Soil Texture’ (20%) being positively associated with coarser textured soils (Supplementary Table S9).

We then estimated the effects of the two climatic and the two edaphic (*T*_min_, *P*_dq_, Soil Nutrient Status, and Soil Texture) gradients on the environmental component of each measured trait using partial Kendall’s *τ* (Table 3). This shows that drier conditions (lower *P*_dq_) were associated with higher *LMA*, *LDMC, C*_m_, *K*_m_, and ρ*_W_* and lower *L*_a_. Higher *T*_min_ was negatively related to *P*_m_. Soil nutrient status seems to be positively associated with higher ρ_W_ and lower *P*_m_. Sandier soils were also inferred to lead to increased *C*_m_ but lower *K*_m_. We note that these trait-environment relationships express the cross-species, ‘pure’ environmental-driven trait variation, since the effect of leaf habit and taxonomy were removed from this analysis. The same partial correlation analysis was performed using the raw plot level average trait values (Supplementary Table S10). Most of the significant partial correlations were common between the environmental component and the average plot data (Supplementary Figure S3), with the exception of the *L*_a_ – *P*_dq_ and the ρ_W_ – *P*_dq_ and ρ_W_ – Soil Nutrient Status association.

**TABLE 3:** Partial Kendall correlation coefficients between the environmental component of each traits’ variation and the four axes of environmental variation across the MEDIT plot network.

	***T*_min_**	***P*_dq_**	**Soil nutrient status**	**Soil texture**
*L*_a_	0.123	**0.343**	−0.100	−0.078
*LMA*	0.116	−**0.220**	0.043	0.076
*L*_t_	0.044	0.073	0.144	−0.025
*LDMC*	0.145	−**0.323**	−0.053	*0.170*
*C*_m_	0.056	−**0.258**	0.039	**0.194**
*N*_m_	−0.038	*0.179*	−0.102	0.110
*P*_m_	−**0.395**	−0.045	−**0.245**	−0.013
*Ca*_m_	0.085	*0.167*	0.073	−0.034
*Mg*_m_	0.017	−0.061	−0.105	*0.181*
*K*_m_	−0.131	−**0.231**	−0.038	−**0.247**
*A*_sat,a_	0.077	0.010	−0.065	−0.017
*R*_dark,a_	0.026	−0.158	0.079	−0.061
ρ_W_	−0.026	−**0.214**	**0.227**	0.060

*Bold values indicate statistically significant associations (*p* < 0.05), after controlling for the effect of all other environmental dimensions, and italic indicate marginally significant associations (*p* < 0.1). Trait abbreviations: *L*_*a*_, leaf area; *LMA*, leaf dry mass per area; *LDMC*, leaf dry matter content; *L*_*t*_, leaf thickness; *N*_*m*_ – *P*_*m*_ – *Ca*_*m*_ – *Mg*_*m*_ – *K*_*m*_, leaf N, P, Ca, Mg, and K mass basis concentrations; *A*_*sat,a*_, light saturated photosynthetic rate on area basis; *R*_*dark,a*_, dark respiration rate on area basis; ρ_*w*_, wood density.*

**FIGURE 8:**
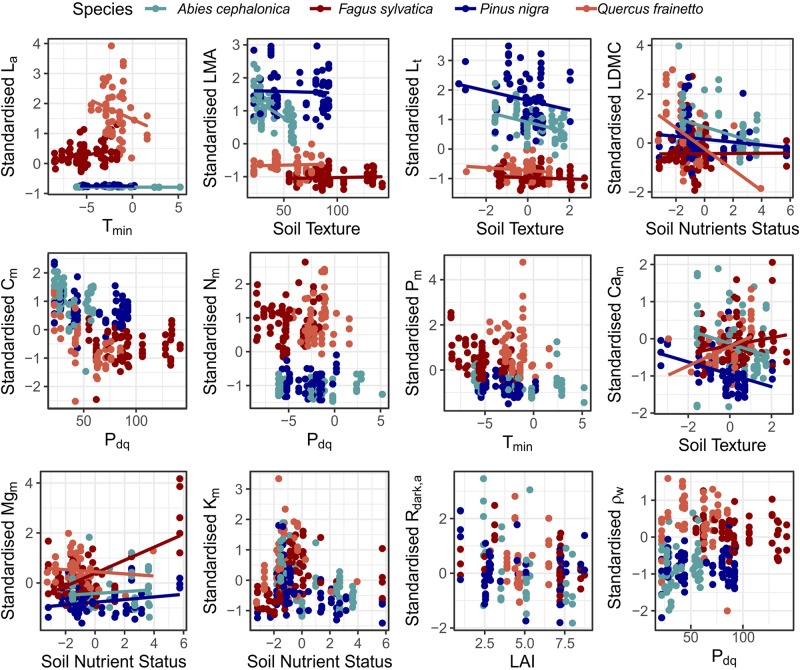
Linear mixed effect models for the measured functional traits, across the major axes of environmental variation identified along the MEDIT forest plot network for the four best-studied species. For traits that regression lines are presented, the analysis suggested that along the respective axis of environmental variation the optimum random structure required different slope for each species (Supplementary Table S11). In cases with no regression lines the optimum random structure required only varying intercepts.

A summary of the linear mixed effect model analysis for the four most widely measured species in our study is presented in Supplementary Table S11, with the inferred relationships shown in Figure 8. For almost half of the traits (*L*_a_, *LMA, L*_t_, *LDMC, Ca*_m_, and *Mg*_m_) the lowest AIC model included random intercepts associated with plot identity (meaning that even after accounting for the independent covariate that there were significant systematic effects of sampling plot on mean values of the trait under investigation) with random intercepts and slopes for species along one of the environmental gradient (suggesting that different species may respond to environmental variations in fundamentally different ways). In particular *L*_a_ responded differently along *T*_min_ variation, *LMA*, *L*_t_ and *Ca*_m_ responded differently along variations in soil texture and *LDMC* and *Mg*_m_ varied individualistic along soil the nutrient status gradient. On the other hand *C*_m_, *N*_m_, *P*_m_, *K*_m_, *A*_sat,a_, *R*_dark,a_, and ρ_W_ were best modelled with just random intercepts for plots and species.

In terms of the fixed effects, an increase in *LAI* positively affected *L*_a_ and negatively *LMA* and *R*_dark,a_. An increase in *T*_min_ was positively associated with *K*_m_ and higher *P*_dq_ had a positive effect on *L*_t_ and a negative effect on *LDMC* and *C*_m_. Soil nutrients availability had a positive effect on *Mg*_m_ and a negative effect on *K*_m_ and finally, coarser soils were associated with higher *L*_a_ and *Mg*_m_. Slope estimates among the four species along the main climatic and edaphic gradients differed, in some cases even in sign (Figure 8), suggesting that although a common trait response to environmental variability can in some cases be identified (see previous paragraph), that there are also species-specific trait-environment relationships which may even trend in opposite directions.

## Discussion

### Plant Functional Types in Mountainous Mediterranean Forests

Our results demonstrate a clear grouping of the studied species to PFTs based on leaf habit, when species average trait values are considered (Figure 1). This reaffirms the *a priori Ne*, *Be*, and *Bd* classification on MMF when ‘approached’ from a trait-based perspective. Our multivariate analysis of species average trait values further identified three functional dimensions in support of the Leaf-Height-Seed (LHS) framework (Westoby, 1998). The first axis (Figure 3 and Table 2) expresses a leaf economic spectrum and contrasts species with cheap leaf construction against species with expensive leaf construction costs (Reich et al., 1997; Kazakou et al., 2007). Interestingly, *A*_sat,a_ is not associated with cheaper construction-cost leaves in agreement to the notion that variations in leaf photosynthetic capacity may occur more or less independently of variations in construction cost/leaf longevity (Lloyd et al., 2013), as is also suggested by subsequent studies that have found leaf structure, gas exchange and hydraulic traits to be effectively decoupled (Li et al., 2015; Costa-Saura et al., 2016; Rosas et al., 2019). The second functional dimension expresses a maximum adult stature vs. wood density trade-off, that contrasts tall species (that also have higher leaf maintenance cost) against shorter species with a persistent life style. Taller species are better competitors for light (Westoby et al., 2002) and *H*_max_ has been associated with higher growth rates in both tropical (Poorter et al., 2008) and Mediterranean (Martínez-Vilalta et al., 2010) forests, at least in terms of wood volume increment. On the other hand, higher wood density is related to lower growth rates (Enquist et al., 1999), higher survival/endurance (Martínez-Vilalta et al., 2010) and resistance to drought-induced xylem cavitation (Hacke et al., 2001). The third dimension mainly relates to seed mass, expressing a trade-off between seed production and establishment rate (Westoby et al., 2002), and interestingly with leaf calcium concentrations showing a negative association with this third trait dimension, as was also found for tropical trees in the Amazon Basin study of Patiño et al. (2012). In this trait space, *Ne* display high leaf-construction costs and adult stature, *Bd* low leaf-construction costs along a range of *L*_a_ and ρ_W_, and *Be* a relatively high-leaf cost with a generally high ρ_W_ and low *H*_max_ (Figure 3). Overall our analysis supports the existence of the LHS framework in MMF. Nevertheless, this conclusion contrasts with the study of de la Riva et al. (2016), who, working across 38 Mediterranean woody species found no evidence for the LHS orthogonal dimensions. This discrepancy may arise from the taxonomically wider set of species measured in our study (including needleleaf evergreens) allowing for more pronounced inter-species contrasts.

### Differences Between PFTs

For most of the studied foliar properties, *Ne* were found at the conservative region of the trait spectrum (Figure 3 and Supplementary Table S4), in agreement to the ‘slow seedling’ hypothesis that has been used to explain *Ne* exclusion from faster growing broadleaved species in productive habitats (Bond, 1989; Brodribb et al., 2012). For example *Ne* species had higher mean *LMA* and lower *N*_m_ and *P*_m_ than *Bd* species, yielding a lower mass-based photosynthetic and respiration rate (with differences eliminated when expressed on an area basis) as also reported in other studies (Lusk et al., 2003). These photosynthetic differences might be attributable to the higher hydraulic capacity and stomatal conductance of angiosperms that enables them to sustain higher transpiration rates (Lusk et al., 2003; Brodribb et al., 2005). As expected, *Ne* were characterised by the lowest ρ_W_ between the three PFTs due to their simpler wood structure consisting mainly from tracheids in contrast to the more complex angiosperm wood structure (Zhang et al., 2017). The wood anatomical differences are considered part of a suite of traits that form two generic hydraulic strategies with gymnosperms operating on safer hydraulic margins and having higher cavitation resistance and lower xylem recovery capacity in contrast to angiosperms (Choat et al., 2012; Carnicer et al., 2013). However recent studies show PFT classifications may not capture hydraulic differences (Anderegg, 2015) with the severity of drought being a stronger predictor of tree mortality than PFT grouping (Greenwood et al., 2017). Among the studied angiosperms a continuum of ‘*fast vs. slow’* plant strategies (Reich, 2014) seems to emerge, with more conservative trait values observed for *Be* compared to *Bd* species. For example, *LMA*, *L*_t_, *C*_m_ were higher and *N*_m_, *P*_m_, *Mg*_m_, *A*_sat,m_, *R*_dark,m_, and ρ_W_ were significantly lower in the studied *Be* species compared to their *Bd* counterparts. In tropical and temperate forests a potential coordination of gas exchange and hydraulic architecture has been reported (Brodribb and Feild, 2000; Maherali et al., 2006; Zhu et al., 2013), with ρ_W_ found to negatively correlate with hydraulic conductivity and photosynthetic rates (Santiago et al., 2004; Hoeber et al., 2014). In addition ρ_W_ has been proposed as a proxy for cavitation vulnerability with denser wood species showing higher cavitation resistance (Hacke et al., 2001; Rosner, 2017). The above seems to agree with the more conservative and drought resistant strategy followed by Mediterranean *Be* species that also seems to explain their occurrence at drier environmental conditions (Costa-Saura et al., 2016).

Most of the broadleaf evergreen species (*Arbutus unedo*, *Arbutus andrachnae*, *Quercus coccifera*, *Quercus ilex*, and *Phillyrea latifolia)* and some of the broadleaf deciduous species (*Fraxinus ornus, Ostrya carpinifolia*, and *Pistacia terebinthus)* in this study are commonly found in the understory of MMF. Functional traits such as *LMA* are known to be sensitive to variation in light availability, with higher irradiance leading to higher *LMA* (Poorter et al., 2009). Intraspecific *LMA* variability between sunlit and shaded leaves has been shown for all three PFTs (Wyka et al., 2012; Gratani, 2014) with lower values recorded in shaded leaves and potentially leading to downregulation of gas exchange rates (Chen et al., 2014). In our study, all understory species that had no individual at the sunlit part of the canopy were sampled for trait measurements outside the plot, in sunnier places in order to reduce such environmental effects and enable between species and sites comparisons. However such *LMA* values will not be representative of the conditions experienced by individuals in the understory of our stands, and could for example lead to overestimation of *A*_sat_ and/or *R*_dark_.

Our analysis found evidence for differences and similarities in multi-trait coordination when the three PFTs were considered separately (Figure 4 and Supplementary Table S6). In *Ne* the first functional dimension (Figure 4B) reveals a needle economic spectrum, with bigger needles characterised by a higher dry mass per area, thickness and light saturated photosynthetic rate (Oren et al., 1986). The first *Bd* dimension is similar to that of *Ne*, with *R*_dark,a_ contributing stronger than *A*_sat,a_ though (Figure 4C). Interestingly, as also documented in tropical tree species (Patiño et al., 2012), *Ca*_m_ is negatively associated with PC1 for broadleaf deciduous species, suggesting a leaf construction cost dimension. In particular leaves with low *LMA* and high mineral content might emerge from thinner, less lignified cell walls and potentially associated with higher levels of organic acid (Poorter and de Jong, 1999). The second *Ne* and *Bd* dimensions integrate leaf and wood traits, with species of higher leaf and wood tissue density (*LDMC* and ρ_W_) characterised by lower leaf nutrient concentrations, and highlight *LDMC* as a better indicator of resource capture and use strategy than *LMA* (Wilson et al., 1999; Hodgson et al., 2011). This resource use dimension seems to explain community dynamics with high (leaf and wood) tissue density (conservative) species exhibiting higher resistance to physical damage, higher drought tolerance and survival compared to low tissue density species (Markesteijn et al., 2011; Lasky et al., 2014). In *Be* species on the other hand, an integrated leaf and wood dimension emerged in PC1 (Figure 4D) indicating trait converge in a previously documented plant economic spectrum (de la Riva et al., 2016). At the ‘fast turnover’ end of the spectrum there are plants with relatively low *LMA*, *LDMC*, ρ_W_, and high *L*_a_ and nutrient/cations contents in contrast to the ‘slow turnover’ end. However as in the across PFTs analysis, the fast-slow turnover dimension identified in the broadleaf evergreen type seems to be independent to the gas exchange axis identified in PC2, where *A*_sat,a_ and *R*_dark,a_ seem to trade-off with *Ca*_m_. The different pathways leaf and wood economics are realised between PFTs, could potentially relate to the different leaf habit/longevity and/or environmental conditions under which they grow. For example the spatial scale of the analysis might be important, with de la Riva et al. (2016) showing that coordination between functional traits becomes weaker or disappears when considering species belonging to environmentally similar conditions. Thus our results suggest that trait covariance patterns depend strongly on the unit of organisation probed (Anderegg et al., 2018).

Trait inter-correlations are useful to identify functional trade-offs and plant strategies, while at the same time are frequently used in dynamic vegetation models to infer one functional trait from another (Sakschewski et al., 2015). The next generation of dynamic vegetation models represent functional diversity within traits’ spectra, rather than mean species or mean PFT values (Scheiter et al., 2013; Fyllas et al., 2014), and thus universal scaling relationships are particularly useful. Nevertheless, recent studies suggest that many trait covariances may not hold at local spatial scales (Messier et al., 2017; Anderegg et al., 2018). We found differences in some key trait-pair scaling relationships between PFTs in MMF (Supplementary Table S7). For example we found a negative relationship among *LMA* and *L*_a_ in the full dataset and within *Be*, but with positive relationship found for *Ne* (Figure 5J). In the *Ne* case this may be attributable to bigger needles being also thicker, with previous studies showing that the strength of the *LMA* – *L*_a_ association was lost when considered within gymnosperms (Ackerly and Reich, 1999). In our study ρ_W_ scaled positively with *LMA* across angiosperms (both *Bd* and *Be*), suggesting a potential coordination of leaf and wood traits (Santiago et al., 2004; Chave et al., 2009; Patiño et al., 2012), in accordance with the coordination of these two traits within *Fagaceae* (Vilà-Cabrera et al., 2015). Across PFTs though a negative association was found (Figure 5L), in agreement with the findings of Vilà-Cabrera et al. (2015) that included both members from *Pinaceae* and *Fagaceae*. However, a consistent positive *LDMC* – ρ_W_ relationship was observed across all species and PFTs (Figure 5B), highlighting the use of *LDMC* as a potentially better surrogate for component leaf traits than *LMA* (Richardson et al., 2013). Interestingly, the negative relationship among *A*_sat,a_ and *LMA* across PFTs is actually positive when considered within *Bd* and *Ne* species separately (Figure 6D). This shift could be explained by the contrasting sources of *LMA* variation that could lead to different relationships between *A*_sat,a_ (Osnas et al., 2018). Thus across PFTs, where *LMA* variation relates to structural toughness and leaf longevity, a negative association is expected. These differences highlight that the use of global scaling relationships could be problematic when parameterising dynamic vegetation models, particularly at regional and local scales. We therefore suggest that PFT specific parameterisations need to be developed so as to better represent trait covariation relationships that are usually embedded in such models (Fyllas et al., 2017a).

### Functional Trait Variation Along Environmental Gradients

Across our dataset *L*_a_ was the most variable trait followed by *LMA*, *R*_dark,a_, and *L*_t_. Various evolutionary, ontogenetic and environmental effects control variation in *LMA* (Poorter et al., 2009), which may be best considered as a part of an integrated trait complex (Poorter et al., 2014). In this study, variation in the three leaf structural traits (*L*_a_, *LMA*, *L*_t_) occurred mainly between PFTs, indicating a certain degree of trait conservatism within these groups (Figure 7). Similar findings were reported for *LMA* and *N*_m_ between angiosperms and gymnosperms in Spain (Vilà-Cabrera et al., 2015). Wood density, on the other hand, was rather ‘stable,’ although again most of the variation was found at the PFT level (Vilà-Cabrera et al., 2015). In terms of leaf elemental concentrations, *C*_m_, *N*_m_, and *P*_m_ were the least variable nutrients (de la Riva et al., 2018). Higher variability in *K*_m_, *Ca*_m_, and *Mg*_m_ suggests that these macronutrients are probably scarce across our MMF plots network, in accordance to the ‘hypothesis of the stability of the limiting elements’ that postulates that limiting nutrients are less variable than more abundant ones (Han et al., 2011). Additionally variation in some nutrients such as Ca and Mg, could also be explained by the high variation in pH and amount of calcareous rocks across our sites, that is known to have a strong influence on the ability of plants to absorb some cations (Goulding, 2016). At the same time, different patterns of variation among scales were observed between the measured macronutrients, indicating different processes of nutrient regulation across our plots network.

For most of the measured traits climatic effects were stronger predictors of environmental variation than edaphic effects (Table 2). Drier summer conditions lead to increased *LMA*, *LDMC*, *C*_m_, *K*_m_, and ρ_W_ (Supplementary Figure S3), i.e., more conservative leaf and wood deployment. One plant adaption to drier conditions is to reduce the surface area from which they lose water (Givnish, 1987), as also shown in the current and previous studies (Costa-Saura et al., 2016; Rosas et al., 2019). According to Nardini et al. (2014), the reduction in *L*_a_ could lead to higher vein density and thus higher *LMA*, offering drought tolerance in Mediterranean plants. It should be noted thought that trait-specific shifts with environmental variables might be better viewed as a coordinated response, especially for traits that have a high number of linkages with other traits, such as *LMA* (Nardini et al., 2014; Costa-Saura et al., 2016). At the same time, ρ_W_ is known to increase with water deficit across and within species (Preston et al., 2008; Patiño et al., 2012) and even intra-annually within the same individual (Bouriaud et al., 2005). Wood density is related to minimum leaf water potential (Ackerly, 2004; Bucci et al., 2004), cavitation resistance (Hacke et al., 2001) and lower mortality rates (Martínez-Vilalta et al., 2010) and thus plants with high ρ_W_ should, generally speaking, found toward the conservative end of the *fast-slow* spectrum (Reich, 2014). We also note that all the environmental – trait associations reported here refer to the true environmental effect on trait variability, after removing variation attributed to the taxonomic and/or PFT classification, and thus refer to adaptive trait response to changes in growing conditions.

The trait analysis of the four most common species in our plots network revealed that different environmental variables control intraspecific trait variation, leading to trait-specific and idiosyncratic species responses. For example denser stands (higher *LAI*) had a common positive effect on the *LMA* of all four species, but at the same time variation in soil texture affected interspecific *LMA* variation of *Abies cephalonica* in a stronger way (Figure 8). In a similar way although an increase in dry season precipitation lead to lower *LDMC* for all species the effect of soil nutrient availability was much stronger for *Quercus frainetto* individuals. However, for some other traits like *C*_m_ it seems that a common response along dry season precipitation can be identified, while for others like *A*_sat,a_ neither PFT classification nor any of the environmental predictors used in this study could adequately capture intraspecific variation. All the above illustrate that traits may respond individualistically within species across some key environmental gradients, sometimes even when comparing within the one PFT.

## Conclusion

In this study we verify differences in key functional traits, between the three most abundant PFTs on Mediterranean Mountain Forests. Multivariate analysis of a set of traits, expressing whole-plant economics, support the existence of the three independent axes as suggested by the LHS framework (Westoby, 1998). However, when foliar and wood trait covariation was examined within each PFT, we found different bivariate associations and different functional dimensions, suggesting that trees within each PFT might optimise their coordinated trait responses in alternative ways (Wyka et al., 2012). Also, some traits showed a greater taxonomic variability than others (*L*_a_, *LMA*, *L*_t_, and *R*_dark,a_ being the most variable) and some other traits such as *K*_m_, *LDMC*, and *R*_dark,a_ were more responsive to environmental variation. Our analysis also shows that drier conditions may lead to more conservative trait syndromes as exemplified by increased *LMA*, *LDMC*, *C*_m_, and ρ_W_. However, when explored within populations of the same species, environmental gradients may drive trait variations in different directions for different species. Our findings highlight the effects of source of variation and local environmental conditions on trait values and trait covariation. We thus suggest the use of regional and local data wherever possible when modelling forest function with trait-based approaches.

## Data Availability Statement

The datasets generated for this study are available on request to the corresponding author.

## Author Contributions

NF and JL conceived the study. NF made the gas exchange measurements and undertook all statistical analyses. CM and AG undertook plant sampling and functional trait measurements. EE and CT undertook soil physical and chemical properties measurements. JZ-C helped with gas exchange measurement protocols. PD, MA, and JL subsequently contributed to the original manuscript drafted from NF. All authors commented and approved the manuscript.

## Conflict of Interest

The authors declare that the research was conducted in the absence of any commercial or financial relationships that could be construed as a potential conflict of interest.

## References

[B1] Ackerly D. (2004). Functional strategies of chaparral shrubs in relation to seasonal water deficit and disturbance. *Ecol. Monogr.* 74 25–44. 10.1890/03-4022

[B2] Ackerly D. D. Reich P. B. (1999). Convergence and correlations among leaf size and function in seed plants: a comparative test using independent contrasts. *Am. J. Bot.* 86 1272–1281. 10.2307/2656775 10487815

[B3] Adler P. B. Salguero-Gómez R. Compagnoni A. Hsu J. S. Ray-Mukherjee J. Mbeau-Ache C. (2014). Functional traits explain variation in plant life history strategies. *Proc. Natl. Acad. Sci. U.S.A.* 111 740–745. 10.1073/pnas.1315179111 24379395 PMC3896207

[B4] Anderegg L. D. L. Berner L. T. Badgley G. Sethi M. L. Law B. E. HilleRisLambers J. (2018). Within-species patterns challenge our understanding of the leaf economics spectrum. *Ecol. Lett.* 21 734–744. 10.1111/ele.12945 29569818

[B5] Anderegg W. R. (2015). Spatial and temporal variation in plant hydraulic traits and their relevance for climate change impacts on vegetation. *New Phytol.* 205 1008–1014. 10.1111/nph.12907 25729797

[B6] Asner G. P. Anderson C. B. Martin R. E. Knapp D. E. Tupayachi R. Sinca F. (2014). Landscape-scale changes in forest structure and functional traits along an Andes-to-Amazon elevation gradient. *Biogeosciences* 11 843–856. 10.5194/bg-11-843-2014

[B7] Atkin O. K. Bloomfield K. J. Reich P. B. Tjoelker M. G. Asner G. P. Bonal D. (2015). Global variability in leaf respiration in relation to climate, plant functional types and leaf traits. *New Phytol.* 206 614–636. 10.1111/nph.13253 25581061

[B8] Berendse F. Scheffer M. (2009). The angiosperm radiation revisited, an ecological explanation for Darwin’s ‘abominable mystery.’. *Ecol. Lett.* 12 865–872. 10.1111/j.1461-0248.2009.01342.x 19572916 PMC2777257

[B9] Bond W. J. (1989). The tortoise and the hare: ecology of angiosperm dominance and gymnosperm persistence. *Biol. J. Linn. Soc.* 36 227–249. 10.1111/j.1095-8312.1989.tb00492.x

[B10] Bouriaud O. Leban J.-M. Bert D. Deleuze C. (2005). Intra-annual variations in climate influence growth and wood density of Norway spruce. *Tree Physiol.* 25 651–660. 10.1093/treephys/25.6.651 15805085

[B11] Bouyoucos G. J. (1962). Hydrometer method improved for making particle size analyses of soils1. *Agron. J.* 54 464–465. 10.2134/agronj1962.00021962005400050028x

[B12] Box E. O. Fujiwara K. (eds) (2015). *Warm-Temperate Deciduous Forests Around the Northern Hemisphere.* Berlin: Springer.

[B13] Bremner J. Mulvaney C. (1982). “Nitrogen-total,” in *Methods of Soil Analysis. Part 2, Chemicaland Microbiological Properties*, eds Page A. L. Miller R. H. Keeney D. R. (Madison, WI: Soil Science Society of America), 595–624.

[B14] Brodribb T. J. Feild T. S. (2000). Stem hydraulic supply is linked to leaf photosynthetic capacity: evidence from New Caledonian and Tasmanian rainforests. *Plant Cell Environ.* 23 1381–1388. 10.1046/j.1365-3040.2000.00647.x

[B15] Brodribb T. J. Holbrook N. M. Zwieniecki M. A. Palma B. (2005). Leaf hydraulic capacity in ferns, conifers and angiosperms: impacts on photosynthetic maxima. *New Phytol.* 165 839–846. 10.1111/j.1469-8137.2004.01259.x 15720695

[B16] Brodribb T. J. Pittermann J. Coomes D. A. (2012). Elegance versus speed: examining the competition between conifer and angiosperm trees. *Int. J. Plant Sci.* 173 673–694. 10.1086/666005

[B17] Bucci S. J. Goldstein G. Meinzer F. C. Scholz F. G. Franco A. C. Bustamante M. (2004). Functional convergence in hydraulic architecture and water relations of tropical savanna trees: from leaf to whole plant. *Tree Physiol.* 24 891–899. 10.1093/treephys/24.8.891 15172839

[B18] Carnicer J. Barbeta A. Sperlich D. Coll M. Peñuelas J. (2013). Contrasting trait syndromes in angiosperms and conifers are associated with different responses of tree growth to temperature on a large scale. *Front. Plant Sci.* 4:409. 10.3389/fpls.2013.00409 24146668 PMC3797994

[B19] Chave J. Coomes D. Jansen S. Lewis S. L. Swenson N. G. Zanne A. E. (2009). Towards a worldwide wood economics spectrum. *Ecol. Lett.* 12 351–366. 10.1111/j.1461-0248.2009.01285.x 19243406

[B20] Chen A. Lichstein J. W. Osnas J. L. D. Pacala S. W. (2014). Species-independent down-regulation of leaf photosynthesis and respiration in response to shading: evidence from six temperate tree species. *PLoS One* 9:e91798. 10.1371/journal.pone.0091798 24727745 PMC3984078

[B21] Choat B. Jansen S. Brodribb T. J. Cochard H. Delzon S. Bhaskar R. (2012). Global convergence in the vulnerability of forests to drought. *Nature* 491 752–755. 10.1038/nature11688 23172141

[B22] Costa-Saura J. M. Martínez-Vilalta J. Trabucco A. Spano D. Mereu S. (2016). Specific leaf area and hydraulic traits explain niche segregation along an aridity gradient in Mediterranean woody species. *Perspect. Plant Ecol. Evol. Syst.* 21 23–30. 10.1016/j.ppees.2016.05.001

[B23] de la Riva E. G. de la Tosto A. Pérez-Ramos I. M. Navarro-Fernández C. M. Olmo M. Anten N. P. R. (2016). A plant economics spectrum in Mediterranean forests along environmental gradients: Is there coordination among leaf, stem and root traits? *J. Veg. Sci.* 27 187–199. 10.1111/jvs.12341

[B24] de la Riva E. G. Villar R. Pérez-Ramos I. M. Quero J. L. Matías L. Poorter L. (2018). Relationships between leaf mass per area and nutrient concentrations in 98 Mediterranean woody species are determined by phylogeny, habitat and leaf habit. *Trees* 32 497–510. 10.1007/s00468-017-1646-z

[B25] De Micco V. Aronne G. (2012). “Morpho-anatomical traits for plant adaptation to drought,” in *Plant Responses to Drought Stress: From Morphological to Molecular Features*, ed. Aroca R. (Heidelberg: Springer), 37–61. 10.1007/978-3-642-32653-0_2

[B26] Díaz S. Kattge J. Cornelissen J. H. C. Wright I. J. Lavorel S. Dray S. (2016). The global spectrum of plant form and function. *Nature* 529 167–171. 10.1038/nature16489 26700811

[B27] Doran J. W. Jones A. J. Smith J. L. Doran J. W. (1996). “Measurement and use of pH and electrical conductivity for soil quality analysis,” in *Methods for Assessing Soil Quality*, eds Doran J. W. Jones A. J. (Madison, WI: SSSA Special Publication), 10.2136/sssaspecpub49.c10

[B28] Enquist B. J. West G. B. Charnov E. L. Brown J. H. (1999). Allometric scaling of production and life-history variation in vascular plants. *Nature* 401 907–911. 10.1038/44819

[B29] Fyllas N. M. Bentley L. P. Shenkin A. Asner G. P. Atkin O. K. Díaz S. (2017a). Solar radiation and functional traits explain the decline of forest primary productivity along a tropical elevation gradient. *Ecol. Lett.* 20 730–740. 10.1111/ele.12771 28464375

[B30] Fyllas N. M. Christopoulou A. Galanidis A. Michelaki C. Z. Dimitrakopoulos P. G. Fulé P. Z. (2017b). Tree growth-climate relationships in a forest-plot network on Mediterranean mountains. *Sci. Total Environ.* 598 393–403. 10.1016/j.scitotenv.2017.04.145 28448931

[B31] Fyllas N. M. Gloor E. Mercado L. M. Sitch S. Quesada C. A. Domingues T. F. (2014). Analysing Amazonian forest productivity using a new individual and trait-based model (TFS v.1). *Geosci. Model Dev.* 7 1251–1269. 10.5194/gmd-7-1251-2014

[B32] Fyllas N. M. Patiño S. Baker T. R. Bielefeld Nardoto G. Martinelli L. A. Quesada C. A. (2009). Basin-wide variations in foliar properties of Amazonian forest: phylogeny, soils and climate. *Biogeosciences* 6 2677–2708. 10.5194/bg-6-2677-2009

[B33] Fyllas N. M. Quesada C. A. Lloyd J. (2012). Deriving Plant Functional Types for Amazonian forests for use in vegetation dynamics models. *Perspect. Plant Ecol. Evol. Syst.* 14 97–110. 10.1016/j.ppees.2011.11.001

[B34] Gardner W. H. (1986). “Water content,” in *Methods of Soil Analysis: Part 1—Physical and Mineralogical Methods*, 2nd Edn, ed. Klute A. (Madison, WI: SSSA), 493–544. 10.2136/sssabookser5.1.2ed.c21

[B35] Garnier E. Navas M.-L. Grigulis K. (2016). *Plant Functional Diversity: Organism Traits, Community Structure, and Ecosystem Properties.* Oxford: Oxford University Press.

[B36] Givnish T. (2002). Adaptive significance of evergreen vs. deciduous leaves: solving the triple paradox. *Silva Fenn.* 36 703–743. 10.14214/sf.535

[B37] Givnish T. J. (1987). Comparative studies of leaf form: assessing the relative roles of selective pressures and phylogenetic constraints. *New Phytol.* 106 131–160. 10.1111/j.1469-8137.1987.tb04687.x

[B38] Gleason S. M. Westoby M. Jansen S. Choat B. Hacke U. G. Pratt R. B. (2016). Weak tradeoff between xylem safety and xylem-specific hydraulic efficiency across the world’s woody plant species. *New Phytol.* 209 123–136. 10.1111/nph.13646 26378984

[B39] Goulding K. W. T. (2016). Soil acidification and the importance of liming agricultural soils with particular reference to the United Kingdom. *Soil Use Manage.* 32 390–399. 10.1111/sum.12270 27708478 PMC5032897

[B40] Gratani L. (2014). Plant phenotypic plasticity in response to environmental factors. *Adv. Bot.* 2014:208747 10.1155/2014/208747

[B41] Greenwood S. Ruiz-Benito P. Martínez-Vilalta J. Lloret F. Kitzberger T. Allen C. D. (2017). Tree mortality across biomes is promoted by drought intensity, lower wood density and higher specific leaf area. *Ecol. Lett.* 20 539–553. 10.1111/ele.12748 28220612

[B42] Hacke U. G. Sperry J. S. Pockman W. T. Davis S. D. McCulloh K. A. (2001). Trends in wood density and structure are linked to prevention of xylem implosion by negative pressure. *Oecologia* 126 457–461. 10.1007/s004420100628 28547229

[B43] Han W. X. Fang J. Y. Reich P. B. Ian Woodward F. Wang Z. H. (2011). Biogeography and variability of eleven mineral elements in plant leaves across gradients of climate, soil and plant functional type in China. *Ecol. Lett.* 14 788–796. 10.1111/j.1461-0248.2011.01641.x 21692962

[B44] Higgins S. I. Buitenwerf R. Moncrieff G. R. (2016). Defining functional biomes and monitoring their change globally. *Glob. Chang. Biol.* 22 3583–3593. 10.1111/gcb.13367 27207728

[B45] Hodgson J. G. Montserrat-Martí G. Charles M. Jones G. Wilson P. Shipley B. (2011). Is leaf dry matter content a better predictor of soil fertility than specific leaf area? *Ann. Bot.* 108 1337–1345. 10.1093/aob/mcr225 21948627 PMC3197453

[B46] Hoeber S. Leuschner C. Köhler L. Arias-Aguilar D. Schuldt B. (2014). The importance of hydraulic conductivity and wood density to growth performance in eight tree species from a tropical semi-dry climate. *For. Ecol. Manage.* 330 126–136. 10.1016/j.foreco.2014.06.039

[B47] Jin Y. Qian H. (2019). V.PhyloMaker: an R package that can generate very large phylogenies for vascular plants. *Ecography* 42 1353–1359. 10.1111/ecog.04434 PMC9363651 35967255

[B48] Jones J. B. Wolf B. Mills H. A. (1991). *Plant Analysis Handbook: A Practical Sampling, Preparation, Analysis, and Interpretation Guide.* Available at: http://agris.fao.org/agris-search/search.do?recordID=US9326207 (accessed March, 2019).

[B49] José Vidal-Macua J. Ninyerola M. Zabala A. Domingo-Marimon C. Pons X. (2017). Factors affecting forest dynamics in the Iberian Peninsula from 1987 to 2012. The role of topography and drought. *For. Ecol. Manage.* 406 290–306. 10.1016/j.foreco.2017.10.011

[B50] Karger D. N. Conrad O. Böhner J. Kawohl T. Kreft H. Soria-Auza R. W. (2017). Climatologies at high resolution for the earth’s land surface areas. *Sci. Data* 4:170122. 10.1038/sdata.2017.122 28872642 PMC5584396

[B51] Kattge J. Díaz S. Lavorel S. Prentice I. C. Leadley P. Bönisch G. (2011). TRY – a global database of plant traits. *Glob. Chang. Biol.* 17 2905–2935. 10.1111/j.1365-2486.2011.02451.x

[B52] Kazakou E. Garnier E. Navas M.-L. Roumet C. Collin C. Laurent G. (2007). Components of nutrient residence time and the leaf economics spectrum in species from Mediterranean old-fields differing in successional status. *Funct. Ecol.* 21 235–245. 10.1111/j.1365-2435.2006.01242.x

[B53] Lasky J. R. Uriarte M. Boukili V. K. Chazdon R. L. (2014). Trait-mediated assembly processes predict successional changes in community diversity of tropical forests. *Proc. Natl. Acad. Sci. U.S.A.* 111 5616–5621. 10.1073/pnas.1319342111 24706791 PMC3992673

[B54] Legendre P. Legendre L. F. J. (1998). *Numerical Ecology.* Amsterdam: Elsevier Science.

[B55] Li L. McCormack M. L. Ma C. Kong D. Zhang Q. Chen X. (2015). Leaf economics and hydraulic traits are decoupled in five species-rich tropical-subtropical forests. *Ecol. Lett.* 18 899–906. 10.1111/ele.12466 26108338

[B56] Lira-Martins D. Humphreys-Williams E. Strekopytov S. Ishida F. Y. Quesada C. A. Lloyd J. (2019). Tropical tree branch-leaf nutrient scaling relationships vary with sampling location. *Front. Plant Sci.* 10:877. 10.3389/fpls.2019.00877 31333710 PMC6625373

[B57] Lloyd J. Bloomfield K. Domingues T. F. Farquhar G. D. (2013). Photosynthetically relevant foliar traits correlating better on a mass vs an area basis: Of ecophysiological relevance or just a case of mathematical imperatives and statistical quicksand? *New Phytol.* 199 311–321. 10.1111/nph.12281 23621613

[B58] Lusk C. H. Wright I. Reich P. B. (2003). Photosynthetic differences contribute to competitive advantage of evergreen angiosperm trees over evergreen conifers in productive habitats. *New Phytol.* 160 329–336. 10.1046/j.1469-8137.2003.00879.x 33832183

[B59] Maherali H. Moura C. F. Caldeira M. C. Willson C. J. Jackson R. B. (2006). Functional coordination between leaf gas exchange and vulnerability to xylem cavitation in temperate forest trees. *Plant Cell Environ.* 29 571–583. 10.1111/j.1365-3040.2005.01433.x 17080608

[B60] Markesteijn L. Poorter L. Bongers F. Paz H. Sack L. (2011). Hydraulics and life history of tropical dry forest tree species: coordination of species’ drought and shade tolerance. *New Phytol.* 191 480–495. 10.1111/j.1469-8137.2011.03708.x 21477008

[B61] Martínez-Vilalta J. Mencuccini M. Vayreda J. Retana J. (2010). Interspecific variation in functional traits, not climatic differences among species ranges, determines demographic rates across 44 temperate and Mediterranean tree species. *J. Ecol.* 98 1462–1475. 10.1111/j.1365-2745.2010.01718.x

[B62] McGill B. J. Enquist B. J. Weiher E. Westoby M. (2006). Rebuilding community ecology from functional traits. *Trends Ecol. Evol.* 21 178–185. 10.1016/j.tree.2006.02.002 16701083

[B63] Messier J. Lechowicz M. J. McGill B. J. Violle C. Enquist B. J. (2017). Interspecific integration of trait dimensions at local scales: the plant phenotype as an integrated network. *J. Ecol.* 105 1775–1790. 10.1111/1365-2745.12755

[B64] Mitrakos K. (1980). A theory for Mediterranean plant life. *Acta Oecol. Plant.* 1 1245–1252.

[B65] Nardini A. Lo Gullo M. A. Trifilò P. Salleo S. (2014). The challenge of the Mediterranean climate to plant hydraulics: responses and adaptations. *Environ. Exp. Bot.* 103 68–79. 10.1016/j.envexpbot.2013.09.018

[B66] Nelson D. W. Sommers L. E. (1982). “Total carbon, organic carbon, and organic matter 1,” in *Methods of Soil Analysis: Part 2. Chemical and Microbiological Properties Agronomymonogra*, eds Page A. L. Miller R. H. Keeney D. R. (Madison, WI: Soil Science Society of America), 539–579. 10.2134/agronmonogr9.2.2ed.c29

[B67] Nelson R. E. (1982). “Carbonate and Gypsum,” in *Methods of Soil Analysis. Part 2: Chemical and Microbiological Properties*, ed. Page A. L. (Madison, WI: Soil Science Society of America), 181–197.

[B68] Ordoñez J. C. Bodegom P. M. V. Witte J.-P. M. Wright I. J. Reich P. B. Aerts R. (2009). A global study of relationships between leaf traits, climate and soil measures of nutrient fertility. *Glob. Ecol. Biogeogr.* 18 137–149. 10.1111/j.1466-8238.2008.00441.x

[B69] Oren R. Schulze E.-D. Matyssek R. Zimmermann R. (1986). Estimating photosynthetic rate and annual carbon gain in conifers from specific leaf weight and leaf biomass. *Oecologia* 70 187–193. 10.1007/BF00379238 28311656

[B70] Osnas J. L. D. Katabuchi M. Kitajima K. Wright S. J. Reich P. B. Bael S. A. V. (2018). Divergent drivers of leaf trait variation within species, among species, and among functional groups. *Proc. Natl. Acad. Sci. U.S.A.* 115 5480–5485. 10.1073/pnas.1803989115 29724857 PMC6003520

[B71] Patiño S. Fyllas N. M. Baker T. R. Paiva R. Quesada C. A. Santos A. J. B. (2012). Coordination of physiological and structural traits in Amazon forest trees. *Biogeosciences* 9 775–801. 10.5194/bg-9-775-2012

[B72] Poorter H. de Jong R. (1999). A comparison of specific leaf area, chemical composition and leaf construction costs of field plants from 15 habitats differing in productivity. *New Phytol.* 143 163–176. 10.1046/j.1469-8137.1999.00428.x

[B73] Poorter H. Lambers H. Evans J. R. (2014). Trait correlation networks: a whole-plant perspective on the recently criticized leaf economic spectrum. *New Phytol.* 201 378–382. 10.1111/nph.12547 24117716

[B74] Poorter H. Niinemets Ü Poorter L. Wright I. J. Villar R. (2009). Causes and consequences of variation in leaf mass per area (LMA): a meta-analysis. *New Phytol.* 182 565–588. 10.1111/j.1469-8137.2009.02830.x 19434804

[B75] Poorter L. Wright S. J. Paz H. Ackerly D. D. Condit R. Ibarra-Manríquez G. (2008). Are functional traits good predictors of demographic rates? Evidence from five neotropical forests. *Ecology* 89 1908–1920. 10.1890/07-0207.1 18705377

[B76] Preston K. A. Cornwell W. K. DeNoyer J. L. (2008). Wood density and vessel traits as distinct correlates of ecological strategy in 51 California coast range angiosperms. *New Phytol.* 170 807–818. 10.1111/j.1469-8137.2006.01712.x 16684240

[B77] R Core Team (2019). *R: A Language and Environment for Statistical Computing.* Vienna, AU: R Foundation for Statistical Computing Available at: https://www.R-project.org/

[B78] Reich P. B. (2014). The world-wide ‘fast–slow’plant economics spectrum: a traits manifesto. *J. Ecol.* 102 275–301. 10.1111/1365-2745.12211

[B79] Reich P. B. Walters M. B. Ellsworth D. S. (1997). From tropics to tundra: global convergence in plant functioning. *Proc. Natl. Acad. Sci. U.S.A.* 94 13730–13734. 10.1073/pnas.94.25.13730 9391094 PMC28374

[B80] Reich P. B. Wright I. J. Cavender-Bares J. Craine J. M. Oleksyn J. Westoby M. (2003). The evolution of plant functional variation: traits, spectra, and strategies. *Int. J. Plant Sci.* 164 S143–S164. 10.1086/374368

[B81] Revell L. J. (2012). Phytools: an R package for phylogenetic comparative biology (and other things). *Methods Ecol. Evol.* 3 217–223. 10.1111/j.2041-210X.2011.00169.x

[B82] Richardson S. J. Allen R. B. Buxton R. P. Easdale T. A. Hurst J. M. Morse C. W. (2013). Intraspecific relationships among wood density, leaf structural traits and environment in four co-occurring species of Nothofagus in New Zealand. *PLoS One* 8:e58878. 10.1371/journal.pone.0058878 23527041 PMC3601108

[B83] Rosas T. Mencuccini M. Barba J. Cochard H. Saura-Mas S. Martínez-Vilalta J. (2019). Adjustments and coordination of hydraulic, leaf and stem traits along a water availability gradient. *New Phytol.* 223 632–646. 10.1111/nph.15684 30636323

[B84] Rosner S. (2017). Wood density as a proxy for vulnerability to cavitation: size matters. *J. Plant Hydraul.* 1:e001 10.20870/jph.2017.e001

[B85] Sakschewski B. von Bloh W. Boit A. Rammig A. Kattge J. Poorter L. (2015). Leaf and stem economics spectra drive diversity of functional plant traits in a dynamic global vegetation model. *Glob. Chang. Biol.* 21 2711–2725. 10.1111/gcb.12870 25611734

[B86] Santiago L. S. Goldstein G. Meinzer F. C. Fisher J. B. Machado K. Woodruff D. (2004). Leaf photosynthetic traits scale with hydraulic conductivity and wood density in Panamanian forest canopy trees. *Oecologia* 140 543–550. 10.1007/s00442-004-1624-1 15232729

[B87] Scheiter S. Langan L. Higgins S. I. (2013). Next-generation dynamic global vegetation models: learning from community ecology. *New Phytol.* 198 957–969. 10.1111/nph.12210 23496172

[B88] Schrodt F. Domingues T. F. Feldpausch T. R. Saiz G. Quesada C. A. Schwarz M. (2015). Foliar trait contrasts between African forest and savanna trees: genetic versus environmental effects. *Funct. Plant Biol.* 42 63–83. 10.1071/FP14040 32480654

[B89] Siefert A. Violle C. Chalmandrier L. Albert C. H. Taudiere A. Fajardo A. (2015). A global meta-analysis of the relative extent of intraspecific trait variation in plant communities. *Ecol. Lett.* 18 1406–1419. 10.1111/ele.12508 26415616

[B90] Thomas G. W. (1982). “Exchangeable cations,” in *Methods of Soil Analysis: Part 2: Chemical and Microbiological Properties*, eds Page A. L. Miller R. H. Keeney D. R. (Madison, WI: Soil Science Society of America), 159–165. 10.2134/agronmonogr9.2.2ed.c9

[B91] Tjoelker M. G. Oleksyn J. Reich P. B. (2001). Modelling respiration of vegetation: evidence for a general temperature-dependent Q10. *Glob. Chang. Biol.* 7 223–230. 10.1046/j.1365-2486.2001.00397.x

[B92] Turnbull M. H. Griffin K. L. Fyllas N. M. Lloyd J. Meir P. Atkin O. K. (2016). Separating species and environmental determinants of leaf functional traits in temperate rainforest plants along a soil-development chronosequence. *Funct. Plant Biol.* 43 751–765. 10.1071/FP16035 32480501

[B93] Vilà-Cabrera A. Martínez-Vilalta J. Retana J. (2015). Functional trait variation along environmental gradients in temperate and Mediterranean trees. *Glob. Ecol. Biogeogr.* 24 1377–1389. 10.1111/geb.12379

[B94] Westoby M. (1998). A leaf-height-seed (LHS) plant ecology strategy scheme. *Plant Soil* 199 213–227. 10.1023/A:1004327224729

[B95] Westoby M. Falster D. S. Moles A. T. Vesk P. A. Wright I. J. (2002). Plant ecological strategies: some leading dimensions of variation between species. *Annu. Rev. Ecol. Syst.* 33 125–159. 10.1146/annurev.ecolsys.33.010802.150452

[B96] Williams K. Field C. B. Mooney H. A. (1989). Relationships among leaf construction cost, leaf longevity, and light environment in rain-forest plants of the genus piper. *Am. Nat.* 133 198–211. 10.1086/284910

[B97] Williamson G. B. Wiemann M. C. (2010). Measuring wood specific gravity… Correctly. *Am. J. Bot.* 97 519–524. 10.3732/ajb.0900243 21622413

[B98] Wilson P. J. Thompson K. Hodgson J. G. (1999). Specific leaf area and leaf dry matter content as alternative predictors of plant strategies. *New Phytol.* 143 155–162. 10.1046/j.1469-8137.1999.00427.x

[B99] Winemiller K. O. Fitzgerald D. B. Bower L. M. Pianka E. R. (2015). Functional traits, convergent evolution, and periodic tables of niches. *Ecol. Lett.* 18 737–751. 10.1111/ele.12462 26096695 PMC4744997

[B100] Wright I. J. Reich P. B. Westoby M. Ackerly D. D. Baruch Z. Bongers F. (2004). The worldwide leaf economics spectrum. *Nature* 428 821–827. 10.1038/nature02403 15103368

[B101] Wyka T. P. Oleksyn J. Żytkowiak R. Karolewski P. Jagodziński A. M. Reich P. B. (2012). Responses of leaf structure and photosynthetic properties to intra-canopy light gradients: a common garden test with four broadleaf deciduous angiosperm and seven evergreen conifer tree species. *Oecologia* 170 11–24. 10.1007/s00442-012-2279-y 22349756 PMC3422461

[B102] Zhang M. Ji C. Zhu J. Wang X. Wang D. Han W. (2017). Comparison of wood physical and mechanical traits between major gymnosperm and angiosperm tree species in China. *Wood Sci. Technol.* 51 1405–1419. 10.1007/s00226-017-0954-1

[B103] Zhu S.-D. Song J.-J. Li R.-H. Ye Q. (2013). Plant hydraulics and photosynthesis of 34 woody species from different successional stages of subtropical forests. *Plant Cell Environ.* 36 879–891. 10.1111/pce.12024 23057774

[B104] Zuur A. Ieno E. N. Walker N. Saveliev A. A. Smith G. M. (2009). *Mixed Effects Models and Extensions in Ecology with R.* New York, NY: Springer-Verlag.

