# Supplementary material for: Functional Trait Variation Among and Within Species and Plant Functional Types in Mountainous Mediterranean Forests
Source: Front Plant Sci. 2020 Mar 4;11:212. doi: 10.3389/fpls.2020.00212 (PMC7065597; doi:10.3389/fpls.2020.00212)

Supporting Information

Table S1: Variation of key environmental variables across the MEDIT forest plot network.

	Lat	Lon	Elevation (m)	T_A (ºC)	T_{min} (ºC)	T_{max} (ºC)	P_A (mm)	P_{dq} (mm)
				**Climate**				
average			1023	10.1	-2.5	25.2	748	67
min	37.01	20.47	374	5.9	-8.5	21.6	365	14
max	41.48	24.71	1665	15	5.4	30.3	1125	142

	Sand (%)	Clay (%)	pH	EC (μs/cm)	SOM (%)	N (%)	P (%)	K (cmol/kg)	Ca (cmol/kg)	Mg (cmol/kg)	WHC (%)
						**Soils**					
average			6.32	168	2.9	0.16	0.053	0.41	15.02	2.26	63.9
min	28	7	4.6	17	0.2	0.02	0.006	0.08	0.73	0.22	33.76
max	81	43	8.5	565	12	0.9	0.22	1.3	58	15	94.54

Table S2: Maximum tree height (H_{max}) per species as inferred from the 0.99 quantile of individual tree height measurements. For species with less than ten individual tree measurements in our plot network an H_{max} value from the literature was used. Species are grouped according to plant functional type (first *Ne*, second *Be* and third *Bd*).

	Hmax (m)	# trees
Abies borisii	22.5	321
Abies cephalonica	25.3	1014
Picea abies	35.0	Literature
Pinus halepensis	23.0	135
Pinus nigra	27.6	1638
Pinus sylvestris	28.7	100
Arbutus andrachne	6.0	Literature
Arbutus unedo	6.0	Literature
Phillyrea latifolia	4.6	40
Quercus coccifera	7.3	303
Quercus ilex	14.1	174
Acer campestre	20	Literature
Betula pendula	18.5	46
Carpinus orientalis	12.4	491
Castanea sativa	22.9	45
Corylus avellana	8.0	Literature
Cotinus coggygria	6.0	19
Fagus sylvatica	25.9	2264
Fraxinus ornus	10.0	25
Ostrya carpinifolia	11.7	49
Pistacia terebinthus	5.1	18
Quercus cerris	15.8	467
Quercus frainetto	18.9	920
Quercus pubescens	19.8	27

Table S3: Average trait values (and standard error), aggregated at species level for the 24 most dominant species encountered in the MEDIT plot network. Species are grouped according to plant functional type (first *Ne*, second *Be* and third *Bd*). Trait abbreviations: L_a - leaf area, *LMA* - leaf dry mass per area, *LDMC* - leaf dry matter content, L_t – leaf thickness, N_m – P_m – Ca_m – Mg_m – K_m leaf N, P, Ca, Mg and K mass basis concentrations, $A_{sat,a}$ light saturated photosynthetic rate on area basis, $R_{dark,a}$ dark respiration rate on area basis and ρ_w wood density. See Table 1 for units.

	L_a	LMA	LDMC	L_t	N_m	P_m	Ca_m	Mg_m	K_m	$A_{sat,a}$	$R_{dark,a}$	ρ_W
Abies borisii	0.46<br>(0.04)	158.57<br>(9.06)	0.41<br>(0.02)	0.39<br>(0.03)	1.10<br>(0.04)	0.09<br>(0.01)	1.03<br>(0.17)	0.18<br>(0.02)	1.11<br>(0.16)	6.06<br>(1.26)	1.10<br>(0.29)	0.62<br>(0.02)
Abies cephalonica	0.54<br>(0.03)	187.44<br>(8.86)	0.46<br>(0.02)	0.41<br>(0.02)	1.06<br>(0.05)	0.09<br>(0.01)	0.88<br>(0.10)	0.23<br>(0.02)	1.09<br>(0.11)	7.44<br>(0.81)	1.20<br>(0.24)	0.61<br>(0.02)
Picea abies	0.28<br>(0.05)	160.71<br>(16.40)	0.42<br>(0.04)	0.39<br>(0.05)	1.28<br>(0.11)	0.16<br>(0.02)	1.20<br>(0.26)	0.17<br>(0.02)	0.43<br>(0.08)	4.92<br>(-)	2.65<br>(-)	0.58<br>(0.03)
Pinus halepensis	1.22<br>(0.36)	157.49<br>(14.37)	0.47<br>(0.03)	0.32<br>(0.02)	1.26<br>(0.07)	0.08<br>(0.01)	0.78<br>(0.08)	0.27<br>(0.03)	0.94<br>(0.19)	10.87<br>(1.60)	0.80<br>(0.38)	0.66<br>(0.03)
Pinus nigra	1.68<br>(0.09)	225.69<br>(8.65)	0.43<br>(0.02)	0.54<br>(0.03)	1.09<br>(0.05)	0.07<br>(0.00)	0.54<br>(0.05)	0.18<br>(0.01)	0.93<br>(0.09)	10.26<br>(0.63)	1.19<br>(0.12)	0.60<br>(0.01)
Pinus sylvestris	0.82<br>(0.09)	166.52<br>(10.50)	0.39<br>(0.01)	0.42<br>(0.02)	1.60<br>(0.09)	0.13<br>(0.02)	0.68<br>(0.06)	0.15<br>(0.01)	0.47<br>(0.05)	11.08<br>(1.53)	0.93<br>(0.33)	0.55<br>(0.02)
Arbutus andrachne	34.35<br>(9.98)	131.35<br>(17.35)	0.38<br>(0.04)	0.34<br>(0.02)	1.40<br>(0.09)	0.09<br>(0.02)	1.06<br>(0.16)	0.26<br>(0.04)	1.51<br>(0.39)	11.12<br>(4.41)	1.69<br>(0.83)	0.81<br>(0.04)
Arbutus unedo	22.29<br>(3.55)	102.88<br>(14.81)	0.35<br>(0.03)	0.29<br>(0.03)	1.17<br>(0.14)	0.07<br>(0.01)	1.11<br>(0.19)	0.30<br>(0.06)	0.99<br>(0.17)	9.14<br>(2.02)	0.88<br>(0.56)	0.73<br>(0.04)
Phillyrea latifolia	3.93<br>(0.95)	134.74<br>(10.93)	0.47<br>(0.03)	0.28<br>(0.02)	1.36<br>(0.15)	0.07<br>(0.01)	1.18<br>(0.26)	0.22<br>(0.04)	1.09<br>(0.22)	11.21<br>(3.05)	1.12<br>(0.66)	0.84<br>(0.03)
Quercus coccifera	4.30<br>(0.58)	155.22<br>(8.47)	0.51<br>(0.03)	0.32<br>(0.02)	1.19<br>(0.05)	0.07<br>(0.01)	1.00<br>(0.09)	0.22<br>(0.03)	0.96<br>(0.14)	7.90<br>(3.00)	0.99<br>(0.52)	0.89<br>(0.02)
Quercus ilex	10.73<br>(2.26)	138.95<br>(15.01)	0.46<br>(0.05)	0.31<br>(0.05)	1.31<br>(0.11)	0.07<br>(0.01)	0.94<br>(0.25)	0.21<br>(0.04)	0.84<br>(0.18)	9.77<br>(2.06)	0.60<br>(0.19)	0.82<br>(0.05)
Acer campestre	14.92<br>(6.66)	47.81<br>(5.91)	0.38<br>(0.06)	0.13<br>(0.02)	2.14<br>(0.24)	0.13<br>(0.01)	1.36<br>(0.31)	0.35<br>(0.03)	1.32<br>(0.13)	7.98<br>(4.66)	0.76<br>(0.60)	0.74<br>(0.03)
Betula pendula	17.36<br>(3.36)	50.84<br>(4.43)	0.35<br>(0.04)	0.15<br>(0.01)	2.92<br>(0.15)	0.18<br>(0.04)	1.17<br>(0.33)	0.41<br>(0.08)	0.70<br>(0.09)	11.20<br>(3.55)	0.85<br>(0.13)	0.65<br>(0.02)
Carpinus orientalis	10.97<br>(2.95)	44.65<br>(7.25)	0.37<br>(0.03)	0.12<br>(0.03)	2.18<br>(0.21)	0.12<br>(0.02)	1.73<br>(0.14)	0.33<br>(0.06)	1.09<br>(0.38)	7.65<br>(2.29)	0.63<br>(0.21)	0.74<br>(0.03)
Castanea sativa	77.70<br>(14.17)	71.75<br>(13.17)	0.35<br>(0.06)	0.21<br>(0.03)	2.80<br>(0.30)	0.17<br>(0.03)	0.73<br>(0.12)	0.52<br>(0.08)	1.34<br>(0.21)	12.94<br>(1.17)	1.23<br>(0.32)	0.66<br>(0.03)
Corylus avellana	61.06<br>(10.32)	35.16<br>(8.86)	0.32<br>(0.04)	0.11<br>(0.01)	2.45<br>(0.24)	0.17<br>(0.03)	1.46<br>(0.32)	0.52<br>(0.12)	0.90<br>(0.14)	6.42<br>(1.54)	1.37<br>(1.30)	0.56<br>(0.03)
Cotinus coggygria	33.33<br>(11.72)	67.89<br>(12.17)	0.35<br>(0.04)	0.19<br>(0.01)	2.07<br>(0.21)	0.11<br>(0.03)	1.78<br>(0.46)	0.29<br>(0.05)	1.55<br>(0.58)	15.83<br>(3.51)	1.27<br>(0.07)	0.58<br>(0.05)

Fagus sylvatica	31.13 (2.08)	49.70 (2.94)	0.37 (0.01)	0.13 (0.01)	2.32 (0.09)	0.14 (0.01)	0.90 (0.06)	0.30 (0.03)	1.12 (0.10)	10.86 (0.72)	1.14 (0.15)	0.71 (0.01)
Fraxinus ornus	20.55 (7.00)	63.85 (8.47)	0.33 (0.02)	0.19 (0.02)	2.03 (0.16)	0.14 (0.03)	1.34 (0.26)	0.36 (0.06)	1.24 (0.30)	13.69 (2.83)	1.24 (0.46)	0.71 (0.05)
Ostrya carpinifolia	23.92 (2.76)	53.77 (4.32)	0.41 (0.03)	0.13 (0.01)	2.25 (0.15)	0.13 (0.01)	1.57 (0.22)	0.33 (0.04)	1.31 (0.34)	11.64 (1.51)	0.99 (0.24)	0.72 (0.02)
Pistacia terebinthus	12.18 (4.49)	84.54 (13.69)	0.41 (0.06)	0.21 (0.03)	2.20 (0.30)	0.13 (0.03)	1.18 (0.20)	0.33 (0.09)	1.19 (0.49)	13.37 (9.44)	1.12 (1.26)	0.79 (0.02)
Quercus cerris	39.28 (4.36)	67.20 (4.67)	0.40 (0.03)	0.17 (0.01)	2.35 (0.14)	0.16 (0.02)	0.79 (0.10)	0.26 (0.02)	1.24 (0.22)	11.32 (2.24)	1.72 (0.39)	0.76 (0.02)
Quercus frainetto	71.92 (6.71)	75.42 (4.49)	0.44 (0.03)	0.17 (0.01)	2.35 (0.11)	0.16 (0.01)	0.86 (0.07)	0.34 (0.02)	1.41 (0.14)	12.08 (1.44)	1.40 (0.20)	0.75 (0.02)
Quercus pubescens	24.62 (4.72)	84.22 (7.24)	0.45 (0.03)	0.19 (0.01)	2.18 (0.12)	0.12 (0.02)	1.05 (0.13)	0.33 (0.05)	0.97 (0.17)	13.66 (2.58)	1.06 (0.16)	0.79 (0.02)

Table S4: Average trait values ± one standard deviation for the three different plant functional types in Mediterranean Forests. Different letters indicate statistically significant differences in the mean trait value between PFTs. Trait abbreviations: L_a - leaf area, *LMA* - leaf dry mass per area, *LDMC* - leaf dry matter content, L_t – leaf thickness, N_m – P_m – Ca_m – Mg_m – K_m leaf N, P, Ca, Mg and K mass basis concentrations, $A_{sat,a}$ light saturated photosynthetic rate on area basis, $R_{dark,a}$ dark respiration rate on area basis and ρ_w wood density.

	**Needleleaf Evergreens**	**Broadleaf Deciduous**	**Broadleaf Evergreens**
L_a (cm^2)	1.00±0.62(a)	40.19±26.58(b)	11.64±11.69(b)
LMA ($g\ m^{-2}$)	189.8±44.2(b)	61.9±20.1(a)	138.6±31.9(b)
LDMC ($g\ g^{-1}$)	0.44±0.07(a)	0.39±0.09(a)	0.46±0.05(a)
L_t (mm)	0.44±0.12(b)	0.16±0.05(a)	0.31±0.06(b)
C_m (%)	47.33±1.29(b)	44.76±1.19(a)	47.00±1.49(b)
N_m ($mg\ g^{-1}$)	1.16±0.25(a)	2.34±0.43(b)	1.25±0.22(a)
N_a($mg\ m^{-2}$)	218.6±62.8(b)	143.9±53.5(a)	172.4±45.3(a,b)
P_m ($mg\ g^{-1}$)	0.09±0.03(b)	0.15±0.04(c)	0.07±0.03(a)
P_a ($mg\ m^{-2}$)	16.40±5.74(b)	8.91±3.74(a)	9.72±3.51(a)
Ca_m ($mg\ g^{-1}$)	0.76±0.36(a)	1.04±0.44(a)	1.05±0.36(a)
Mg_m ($mg\ g^{-1}$)	0.20±0.07(a)	0.34±0.12(b)	0.24±0.09(a)
K_m ($mg\ g^{-1}$)	0.93±0.45(a)	1.21±0.54(a)	1.04±0.48(a)
$A_{sat,a}$ ($\mu mol\ m^{-2}\ s^{-1}$)	9.21±3.09(a)	11.55±3.88(a)	9.51±4.28(a)
$R_{dark,a}$ ($\mu mol\ m^{-2}\ s^{-1}$)	1.15±0.61(a)	1.20±0.59(a)	1.06±0.82(a)
$A_{sat,m}$ ($nmol\ g^{-1}\ s^{-1}$)	46.69±17.15(a)	194.41±70.51(c)	76.90±40.74(b)
$R_{dark,m}$ ($nmol\ g^{-1}\ s^{-1}$)	5.71±0.26(a)	19.64±0.70(b)	8.33±1.08(a)
ρ_W ($g\ cm^{-3}$)	0.61±0.06(a)	0.72±0.07(b)	0.84±0.09(c)

Table S5: Average trait values ± one standard deviation for the two broadleaf plant functional types in Mediterranean Forests. The average values are different to Table S4 because they are calculated as the average of species mean values across sites. Different letters indicate statistically significant differences in the mean trait value between PFTs, after accounting for their phylogenetic history. Trait abbreviations: L_a - leaf area, *LMA* - leaf dry mass per area, *LDMC* - leaf dry matter content, L_t – leaf thickness, N_m – P_m – Ca_m – Mg_m – K_m leaf N, P, Ca, Mg and K mass basis concentrations, $A_{sat,a}$ light saturated photosynthetic rate on area basis, $R_{dark,a}$ dark respiration rate on area basis, ρ_w wood density, H_{max} maximum tree height and S_m seed mass.

	**Broadleaf Deciduous**	**Broadleaf Evergreens**
L_a (cm^2)	33.76±22.61(a)	15.12±13.06(a)
LMA (g m^{-2})	61.3±15.6(a)	132.6±19.0(b)
LDMC (g g^{-1})	0.38±0.04(a)	0.44±0.06(a)
L_t (mm)	0.16±0.04(a)	0.31±0.02(b)
C_m (%)	44.54±0.62(a)	47.01±0.73(b)
N_m (mg g^{-1})	2.33±0.26(b)	1.29±0.10(a)
N_a(mg m^{-2})	141.7±37.3(a)	168.8±29.8(a)
P_m (mg g^{-1})	0.14±0.02(b)	0.07±0.01(a)
P_a (mg m^{-2})	8.69±2.25(a)	9.39±1.81(a)
Ca_m (mg g^{-1})	1.22±0.35(a)	1.06±0.09(a)
Mg_m (mg g^{-1})	0.36±0.08(b)	0.24±0.04(a)
K_m (mg g^{-1})	1.18±0.23(a)	1.08±0.26(a)
$A_{sat,a}$ (μmol m^{-2} s^{-1})	11.43±2.70(a)	9.83±1.39(a)
$R_{dark,a}$ (μmol m^{-2} s^{-1})	1.13±0.29(a)	1.05±0.40(a)
$A_{sat,m}$ (nmol g^{-1} s^{-1})	194.2±36.5(b)	82.9±25.9(a)
$R_{dark,m}$ (nmol g^{-1} s^{-1})	18.3±4.6(b)	8.6±3.9(a)
ρ_W (g cm^{-3})	0.70±0.07(a)	0.82±0.06(b)
H_{max} *(m)*	15.00±6.65(a)	7.60±3.79(a)
S_m *(g)*	1.43±2.85(a)	0.82±1.12(a)

Table S6: Principal Component Analysis of 12 foliar and 1 wood trait, within different plant functional types. Variables that contribute more than the radius of the equilibrium contribution circle (r=0.48), at each principal component (PC) are indicated with bold. Trait abbreviations: L_a - leaf area, *LMA* - leaf dry mass per area, *LDMC* - leaf dry matter content, L_t – leaf thickness, N_m – P_m – Ca_m – Mg_m – K_m leaf N, P, Ca, Mg and K mass basis concentrations, $A_{sat,a}$ light saturated photosynthetic rate on area basis, $R_{dark,a}$ dark respiration rate on area basis and ρ_w wood density. See Table S1 for units.

	All			Needleleaf Evergreens			Broadleaf Deciduous			Broadleaf Evergreens		
	PCA1	PCA2	PCA3	PC1	PC2	PC3	PC1	PC2	PC3	PC1	PC2	PC3
Eigenvalue	5.84	1.53	1.27	3.00	2.19	1.49	2.82	2.67	1.34	3.95	1.97	1.92
Variance (%)	44.91	11.78	9.74	23.10	16.83	11.44	21.71	20.51	10.33	30.35	15.16	14.74
L_a	**0.88**	0.05	0.22	**0.83**	0.00	-0.11	0.45	0.23	**-0.48**	**-0.81**	-0.02	0.22
LMA	**-0.94**	0.11	0.04	**0.79**	0.26	0.12	**0.76**	-0.42	0.33	**0.86**	-0.08	0.27
LDMC	-0.42	-0.23	**0.65**	-0.25	**0.64**	0.46	0.09	**-0.74**	-0.21	**0.80**	0.05	-0.29
L_t	**-0.87**	0.22	-0.21	**0.83**	-0.25	-0.23	**0.73**	0.15	0.52	0.31	-0.15	**0.67**
C_m	**-0.79**	0.06	0.05	-0.28	0.44	0.42	0.51	-0.27	-0.43	0.38	0.12	**-0.60**
N_m	**0.89**	0.14	0.03	-0.11	**-0.72**	0.33	0.16	**0.62**	-0.17	-0.47	0.49	-0.40
P_m	**0.76**	0.27	-0.28	-0.45	**-0.64**	0.10	0.19	**0.79**	-0.09	-0.52	0.17	-0.12
Ca_m	**0.52**	-0.45	-0.09	-0.36	-0.25	-0.09	**-0.59**	0.11	**0.52**	0.01	**-0.59**	0.23
Mg_m	**0.73**	0.02	-0.02	-0.35	0.03	0.07	-0.13	0.34	0.29	**-0.66**	-0.13	0.38
K_m	0.46	0.20	-0.27	-0.08	0.29	-0.36	0.22	**0.52**	-0.12	-0.46	-0.20	**-0.54**
$A_{sat,a}$	0.29	**0.57**	0.48	**0.57**	-0.22	0.46	0.42	-0.12	0.29	-0.10	**0.74**	0.40
$R_{dark,a}$	-0.02	**0.79**	0.24	0.24	0.22	**0.60**	**0.75**	0.21	0.16	0.07	**0.80**	0.28
ρ_W	0.40	-0.37	**0.54**	-0.13	**0.54**	-0.43	0.09	**-0.59**	0.02	**0.72**	0.25	-0.05

Table S7: Bivariate relationships between 12 foliar and 1 wood trait. For every Y and X trait pair, the number of individuals included in the analysis (N), Pearson's correlation coefficient (ρ), and p value, are given along with the intercept (α) and slope (β) of the Standardized Major Axis regression (Y=α+βX) on the $\log_{10}$ transformed data. Trait abbreviations: L_a - leaf area, *LMA* - leaf dry mass per area, *LDMC* - leaf dry matter content, L_t – leaf thickness, N_m – P_m – Ca_m – Mg_m – K_m leaf N, P, Ca, Mg and K mass basis concentrations, $A_{sat,a}$ light saturated photosynthetic rate on area basis, $R_{dark,a}$ dark respiration rate on area basis and ρ_w wood density. See Table 1 for units.

		Needleleaf Evergreens					Broadleaf Deciduous					Broadleaf Evergreens					All species					Slope difference	
Y	X	N	R2	p	intr	slope	N	R2	p	intr	slope	N	R2	p	intr	slope	N	R2	p.val	intr	slope	LR	LR.p
LMA	L_a	204	0.335	0.000	2.310	0.328	294	0.007	0.152			62	0.340	0.000	2.385	-0.311	560	0.625	0.000	2.287	-0.347	32.264	**0.000**
LDMC	L_a	195	0.009	0.199			293	0.001	0.630			61	0.385	0.000	-0.109	-0.291	549	0.081	0.000	-0.292	-0.122	14.219	**0.001**
L_t	L_a	195	0.328	0.000	-0.317	0.372	293	0.004	0.263			61	0.000	0.981			549	0.590	0.000	-0.346	-0.312	14.695	**0.001**
ρw	L_a	202	0.001	0.617			290	0.024	0.008	1.093	-0.251	62	0.356	0.000	1.031	-0.221	554	0.200	0.000	0.578	0.125	9.536	**0.008**
C_m	L_a	200	0.011	0.134			292	0.044	0.000	2.582	0.045	62	0.140	0.003	2.695	-0.030	554	0.369	0.000	2.680	-0.022	9.545	**0.008**
N_m	L_a	201	0.000	0.801			293	0.058	0.000	0.962	0.266	62	0.048	0.086			556	0.645	0.000	1.037	0.214	14.138	**0.001**
P_m	L_a	200	0.113	0.000	-0.119	-0.541	290	0.072	0.000	-0.529	0.455	61	0.035	0.151			551	0.283	0.000	-0.162	0.235	7.666	**0.022**
Ca_m	L_a	201	0.184	0.000	0.761	-0.789	293	0.090	0.000	1.980	-0.647	62	0.018	0.297			556	0.062	0.000	0.701	0.270	15.801	**0.000**
Mg_m	L_a	201	0.007	0.229			293	0.021	0.013	-0.270	0.518	62	0.129	0.004	-0.087	0.439	556	0.357	0.000	0.184	0.245	1.715	0.424
K_m	L_a	201	0.000	0.774			292	0.039	0.001	0.032	0.681	62	0.050	0.079			555	0.122	0.000	0.758	0.271	12.890	**0.002**
$A_{sat,a}$	L_a	139	0.185	0.000	0.935	0.628	201	0.020	0.045	0.146	0.584	38	0.002	0.794			378	0.099	0.000	0.771	0.235	0.770	0.680
$R_{dark,a}$	L_a	135	0.000	0.983			188	0.043	0.004	-1.200	0.805	36	0.001	0.882			359	0.003	0.273			12.866	**0.002**
LDMC	*LMA*	219	0.028	0.013	-1.844	0.652	295	0.253	0.000	-1.646	0.695	94	0.446	0.000	-2.184	0.861	608	0.223	0.000	-1.102	0.355	7.781	**0.020**
L_t	*LMA*	219	0.657	0.000	-2.856	1.098	295	0.539	0.000	-2.375	0.885	94	0.305	0.000	-2.155	0.769	608	0.876	0.000	-2.387	0.889	21.665	**0.000**
ρw	*LMA*	226	0.024	0.019	1.930	-0.583	294	0.094	0.000	-0.163	0.499	97	0.236	0.000	-0.854	0.796	617	0.042	0.000	1.488	-0.396	19.100	**0.000**
C_m	*LMA*	224	0.000	0.875			296	0.088	0.000	2.492	0.090	96	0.038	0.056			616	0.443	0.000	2.531	0.065	11.382	**0.003**
N_m	*LMA*	225	0.054	0.000	3.024	-0.868	297	0.030	0.003	2.290	-0.527	97	0.065	0.012	2.461	-0.643	619	0.658	0.000	2.470	-0.630	32.718	**0.000**
P_m	*LMA*	224	0.084	0.000	3.249	-1.468	294	0.033	0.002	1.754	-0.906	96	0.073	0.008	2.763	-1.380	614	0.391	0.000	1.492	-0.735	34.456	**0.000**
Ca_m	*LMA*	225	0.035	0.005	5.532	-2.072	297	0.048	0.000	3.258	-1.280	97	0.004	0.533			619	0.134	0.000	2.505	-0.782	31.022	**0.000**
Mg_m	*LMA*	225	0.088	0.000	3.640	-1.486	297	0.008	0.122			97	0.043	0.042	3.345	-1.411	619	0.339	0.000	1.812	-0.706	20.666	**0.000**
K_m	*LMA*	225	0.001	0.574			296	0.026	0.006	3.437	-1.349	97	0.003	0.624			618	0.094	0.000	2.628	-0.816	29.691	**0.000**
$A_{sat,a}$	*LMA*	147	0.097	0.000	-3.198	1.801	202	0.094	0.000	-1.008	1.152	39	0.000	0.936			388	0.015	0.016	2.275	-0.644	19.276	**0.000**

$R_{dark,a}$	*LMA*	142	0.083	0.001	-8.136	3.535	189	0.202	0.000	-2.731	1.558	37	0.007	0.613			368	0.005	0.169			65.057	**0.000**
L_t	*LDMC*	219	0.194	0.000	-0.985	-1.684	295	0.047	0.000	-1.358	-1.273	94	0.063	0.015	-0.836	-0.893	608	0.017	0.001	0.371	2.504	30.115	**0.000**
ρ_W	*LDMC*	217	0.015	0.070			292	0.169	0.000	1.020	0.724	94	0.223	0.000	1.156	0.909	603	0.043	0.000	1.127	1.106	7.732	**0.021**
C_m	*LDMC*	216	0.050	0.001	2.741	0.179	294	0.036	0.001	2.705	0.129	93	0.082	0.005	2.718	0.129	603	0.131	0.000	2.734	0.183	15.647	**0.000**
N_m	*LDMC*	217	0.038	0.004	0.577	-1.306	295	0.110	0.000	1.042	-0.760	94	0.048	0.033	0.832	-0.741	606	0.158	0.000	0.518	-1.768	43.026	**0.000**
P_m	*LDMC*	216	0.054	0.001	-0.856	-2.137	292	0.144	0.000	-0.394	-1.301	93	0.105	0.002	-0.735	-1.591	601	0.198	0.000	-0.774	-2.033	32.928	**0.000**
Ca_m	*LDMC*	217	0.002	0.497			295	0.001	0.585			94	0.001	0.789			606	0.013	0.006	0.080	-2.197	47.271	**0.000**
Mg_m	*LDMC*	217	0.005	0.310			295	0.030	0.003	-0.113	-1.477	94	0.034	0.075			606	0.069	0.000	-0.377	-1.980	22.966	**0.000**
K_m	*LDMC*	217	0.002	0.470			294	0.080	0.000	0.233	-1.953	94	0.006	0.466			605	0.049	0.000	0.094	-2.296	39.983	**0.000**
$A_{sat,a}$	*LDMC*	144	0.003	0.536			203	0.029	0.015	1.693	1.568	38	0.015	0.465			385	0.000	0.906			11.252	**0.004**
$R_{dark,a}$	*LDMC*	140	0.016	0.139			190	0.017	0.071			36	0.001	0.848			366	0.001	0.554			40.936	**0.000**
L_t	L_t	217	0.052	0.001	0.412	-0.526	291	0.000	0.792			94	0.020	0.173			602	0.100	0.000	0.424	-0.442	29.570	**0.000**
ρ_W	L_t	216	0.011	0.131			293	0.036	0.001	2.732	0.101	93	0.010	0.347			602	0.376	0.000	2.707	0.073	9.685	**0.008**
C_m	L_t	217	0.014	0.079			294	0.005	0.212			94	0.012	0.284			605	0.575	0.000	0.779	-0.708	12.059	**0.002**
N_m	L_t	216	0.023	0.027	-0.547	-1.281	291	0.012	0.059			93	0.000	0.834			600	0.280	0.000	-0.478	-0.823	20.917	**0.000**
P_m	L_t	217	0.022	0.030	0.134	-1.903	294	0.043	0.000	-0.180	-1.448	94	0.019	0.180			605	0.134	0.000	0.404	-0.881	10.602	**0.005**
K_m	L_t	217	0.000	0.840			293	0.002	0.439			94	0.000	0.889			604	0.067	0.000	0.432	-0.922	13.999	**0.001**
$A_{sat,a}$	L_t	144	0.086	0.000	1.452	1.506	202	0.042	0.003	2.037	1.243	38	0.006	0.635			384	0.017	0.011	0.555	-0.712	10.650	**0.005**
$R_{dark,a}$	L_t	140	0.024	0.068			189	0.317	0.000	1.345	1.637	36	0.003	0.754			365	0.009	0.073			47.462	**0.000**
C_m	ρ_W	222	0.000	0.908			293	0.006	0.182			96	0.002	0.679			611	0.019	0.001	2.776	-0.164	6.003	**0.050**
N_m	ρ_W	223	0.040	0.003	1.962	-1.490	294	0.110	0.000	2.105	-1.038	97	0.021	0.159			614	0.014	0.003	0.110	1.581	30.301	**0.000**
P_m	ρ_W	222	0.095	0.000	1.438	-2.493	292	0.103	0.000	1.417	-1.765	96	0.001	0.766			610	0.013	0.005	1.295	-1.835	18.564	**0.000**
Ca_m	ρ_W	223	0.001	0.631			294	0.012	0.056	2.810	-2.527	97	0.002	0.672			614	0.038	0.000	-0.422	1.960	32.931	**0.000**
Mg_m	ρ_W	223	0.029	0.011	-1.281	2.552	294	0.040	0.001	1.948	-2.009	97	0.067	0.010	1.832	-1.772	614	0.019	0.001	-0.833	1.772	11.803	**0.003**
K_m	ρ_W	223	0.013	0.089			293	0.005	0.212			97	0.014	0.250			613	0.012	0.006	-0.431	2.050	24.443	**0.000**
$A_{sat,a}$	ρ_W	146	0.014	0.153			200	0.001	0.657			39	0.003	0.734			385	0.004	0.211			5.176	0.075
$R_{dark,a}$	ρ_W	141	0.003	0.532			187	0.000	0.829			37	0.041	0.232			365	0.002	0.452			29.968	**0.000**
N_m	C_m	225	0.015	0.065			297	0.006	0.193			96	0.035	0.068			618	0.371	0.000	26.759	-9.596	8.967	**0.011**
P_m	C_m	224	0.022	0.028	32.697	-12.252	294	0.000	0.812			95	0.035	0.070			613	0.245	0.000	29.555	-11.091	5.106	0.078
Ca_m	C_m	225	0.013	0.087			297	0.137	0.000	38.417	-14.117	96	0.011	0.315			618	0.137	0.000	32.566	-11.878	10.759	**0.005**
Mg_m	C_m	225	0.000	0.814			297	0.002	0.487			96	0.017	0.203			618	0.175	0.000	28.990	-10.738	1.392	0.499
K_m	C_m	225	0.006	0.255			296	0.008	0.131			96	0.007	0.416			617	0.042	0.000	33.997	-12.395	6.271	**0.043**

$A_{\mathrm{sat,a}}$	C_{m}	144	0.042	0.014	39.473	-14.400	201	0.000	0.818			39	0.004	0.711			384	0.053	0.000	28.819	-10.456	5.690	0.058
$R_{\mathrm{dark,a}}$	C_{m}	139	0.052	0.007	-64.73	24.191	188	0.041	0.005	-47.66	17.990	37	0.020	0.404			364	0.008	0.093			18.020	**0.000**
$\mathrm{P_m}$	N_{m}	225	0.155	0.000	-1.851	1.679	295	0.224	0.000	-2.169	1.706	96	0.174	0.000	-2.526	2.153	616	0.522	0.000	-1.386	1.163	5.504	0.064
$\mathrm{Ca_m}$	N_{m}	226	0.005	0.295			298	0.005	0.206			97	0.024	0.133			621	0.067	0.000	-0.558	1.238	0.632	0.729
$\mathrm{Mg_m}$	N_{m}	226	0.003	0.402			298	0.035	0.001	-2.126	1.935	97	0.007	0.414			621	0.301	0.000	-0.953	1.118	4.456	0.108
$\mathrm{K_m}$	N_{m}	226	0.072	0.000	3.572	-2.518	297	0.002	0.400			97	0.027	0.107			620	0.053	0.000	-0.568	1.292	0.265	0.876
$A_{\mathrm{sat,a}}$	N_{m}	145	0.024	0.064			202	0.001	0.600			39	0.066	0.114			386	0.078	0.000	-0.294	1.047	9.266	**0.010**
$R_{\mathrm{dark,a}}$	N_{m}	140	0.003	0.497			189	0.015	0.091			37	0.015	0.467			366	0.007	0.110			13.780	**0.001**
$\mathrm{Ca_m}$	P_{m}	226	0.029	0.011	0.946	1.421	295	0.004	0.263			96	0.002	0.642			617	0.047	0.000	0.919	1.067	8.018	**0.018**
$\mathrm{Mg_m}$	P_{m}	226	0.001	0.637			295	0.040	0.001	0.336	1.128	96	0.010	0.344			617	0.173	0.000	0.380	0.961	1.756	0.416
$\mathrm{K_m}$	P_{m}	226	0.011	0.122			294	0.081	0.000	0.826	1.498	96	0.085	0.004	1.191	1.249	616	0.073	0.000	0.973	1.113	2.825	0.243
$A_{\mathrm{sat,a}}$	P_{m}	145	0.003	0.493			200	0.007	0.247			39	0.002	0.773			384	0.021	0.004	0.958	0.944	2.991	0.224
$R_{\mathrm{dark,a}}$	P_{m}	140	0.012	0.203			187	0.077	0.000	-0.229	1.658	37	0.001	0.825			364	0.019	0.008	-0.045	1.495	12.574	**0.002**
$\mathrm{Mg_m}$	Ca_{m}	227	0.028	0.012	-0.327	0.717	298	0.078	0.000	-0.286	0.797	97	0.070	0.009	-0.635	0.990	622	0.138	0.000	-0.450	0.903	7.294	**0.026**
$\mathrm{K_m}$	Ca_{m}	227	0.029	0.010	1.794	-1.055	297	0.007	0.141			97	0.000	0.834			621	0.000	0.751			1.520	0.468
$A_{\mathrm{sat,a}}$	Ca_{m}	146	0.046	0.009	1.567	-0.790	202	0.001	0.684			39	0.060	0.134			387	0.000	0.779			13.971	**0.001**
$R_{\mathrm{dark,a}}$	Ca_{m}	141	0.026	0.057			189	0.134	0.000	1.152	-1.145	37	0.120	0.036	3.032	-3.273	367	0.056	0.000	1.163	-1.279	31.885	**0.000**
$\mathrm{K_m}$	Mg_{m}	227	0.021	0.029	0.514	1.471	297	0.005	0.230			97	0.006	0.448			621	0.067	0.000	0.534	1.155	2.673	0.263
$A_{\mathrm{sat,a}}$	Mg_{m}	146	0.003	0.479			202	0.002	0.524			39	0.000	0.947			387	0.030	0.001	0.637	0.878	0.993	0.609
$R_{\mathrm{dark,a}}$	Mg_{m}	141	0.016	0.139			189	0.009	0.193			37	0.001	0.866			367	0.000	0.796			10.244	**0.006**
$A_{\mathrm{sat,a}}$	K_{m}	146	0.078	0.001	1.627	-0.761	202	0.000	0.836			39	0.053	0.159			387	0.000	0.710			6.067	**0.048**
$R_{\mathrm{dark,a}}$	K_{m}	141	0.000	0.978			189	0.095	0.000	-1.236	1.156	37	0.072	0.108			367	0.012	0.037	-1.261	1.255	15.647	**0.000**
$R_{\mathrm{dark,a}}$	$A_{\mathrm{sat,a}}$	147	0.040	0.016	-1.810	1.916	190	0.036	0.009	-1.557	1.527	37	0.354	0.000	-1.954	1.969	374	0.089	0.000	-1.696	1.714	5.460	0.065
$\mathrm{N_a}$	LMA	225	0.472	0.000	-3.310	1.162	297	0.754	0.000	-2.718	1.046	97	0.643	0.000	-2.996	1.042	619	0.638	0.000	-2.015	0.612	3.647	0.161
$\mathrm{P_a}$	$\mathrm{N_a}$	224	0.246	0.000	-0.933	1.297	294	0.577	0.000	-1.061	1.165	96	0.295	0.000	-0.939	1.418	614	0.568	0.000	-0.963	1.287	5.488	0.064
$A_{\mathrm{sat,a}}$	$\mathrm{N_a}$	144	0.132	0.000	1.851	1.398	201	0.097	0.000	1.977	1.093	39	0.021	0.378			384	0.009	0.061			10.127	**0.006**
$R_{\mathrm{dark,a}}$	$\mathrm{N_a}$	139	0.027	0.054			188	0.222	0.000	1.296	1.467	37	0.027	0.330			364	0.049	0.000	1.250	1.617	34.240	**0.000**
$\mathrm{P_a}$	LMA	224	0.143	0.000	-5.251	1.518	294	0.468	0.000	-4.233	1.221	96	0.182	0.000	-5.167	1.469	614	0.470	0.000	-3.556	0.788	9.581	**0.008**
$A_{\mathrm{sat,a}}$	$\mathrm{P_a}$	143	0.015	0.144			199	0.030	0.014	2.935	0.917	39	0.001	0.853			381	0.001	0.610			5.551	0.062
$R_{\mathrm{dark,a}}$	$\mathrm{P_a}$	138	0.004	0.467			186	0.304	0.000	2.563	1.222	37	0.011	0.543			361	0.053	0.000	2.577	1.312	35.703	**0.000**
$A_{\mathrm{sat,m}}$	LMA	147	0.062	0.002	5.698	-1.767	202	0.258	0.000	4.517	-1.273	39	0.277	0.001	5.901	-1.943	388	0.740	0.000	4.493	-1.254	14.494	**0.001**
$A_{\mathrm{sat,m}}$	$\mathrm{N_m}$	144	0.074	0.001	-0.143	1.699	201	0.025	0.026	-1.040	2.420	39	0.173	0.008	-1.919	3.322	384	0.635	0.000	-0.495	2.028	19.076	**0.000**

$A_{sat,m}$	P_m	143	0.004	0.449			199	0.001	0.608			39	0.038	0.232			381	0.340	0.000	1.929	1.823	7.622	**0.022**
$R_{dark,m}$	*LMA*	142	0.000	0.948			189	0.044	0.004	3.766	-1.423	37	0.046	0.204			368	0.445	0.000	3.766	-1.388	66.873	**0.000**
$R_{dark,m}$	N_m	139	0.000	0.915			188	0.048	0.002	-2.428	2.699	37	0.053	0.169			364	0.440	0.000	-1.684	2.192	17.937	**0.000**
$R_{dark,m}$	P_m	138	0.000	0.827			186	0.163	0.000	1.024	1.508	37	0.016	0.455			361	0.334	0.000	0.933	2.020	18.600	**0.000**

Table S8: Coefficients of variation for each studied trait including all (All) studied individuals, or individuals belonging to needleleaf evergreen (*Ne*), broadleaf deciduous (*Bd*) and broadleaf evergreens (*Be*) species. Trait abbreviations: L_a - leaf area, *LMA* - leaf dry mass per area, *LDMC* - leaf dry matter content, L_t – leaf thickness, N_m – P_m – Ca_m – Mg_m – K_m leaf N, P, Ca, Mg and K mass basis concentrations, $A_{sat,a}$ light saturated photosynthetic rate on area basis, $R_{dark,a}$ dark respiration rate on area basis and ρ_w wood density. See Table S1 for units.

	Leaf and wood structural traits					
	L_a	*LMA*	*LDMC*	L_t	ρ_W	
All	1.22	0.55	0.21	0.53	0.15	
Ne	0.62	0.23	0.17	0.27	0.10	
Bd	0.66	0.33	0.22	0.30	0.10	
Be	1.00	0.23	0.22	0.20	0.11	
	Leaf elemental concentrations					
	C_m	N_m	P_m	Ca_m	Mg_m	K_m
All	0.04	0.39	0.44	0.45	0.45	0.48
Ne	0.03	0.22	0.37	0.48	0.37	0.48
Bd	0.03	0.18	0.31	0.42	0.36	0.44
Be	0.03	0.18	0.36	0.35	0.38	0.46
	Leaf fluxes					
	$A_{sat,a}$	$R_{dark,a}$	$A_{sat,m}$	$R_{dark,m}$		
All	0.36	0.54	0.72	0.76		
Ne	0.33	0.54	0.37	0.55		
Bd	0.34	0.49	0.36	0.46		
Be	0.45	0.77	0.53	0.76		

Table S9: Principal Components Analysis on the edaphic variables across the MEDIT plot network (Fig. S1). Variables that contribute more than the radius of the equilibrium contribution circle (r=0.45), at each principal component (PC), are indicated with bold. (WHC: water holding capacity, Sand and Clay are the respective soil concentrations, SOM the soil organic matter, totalN and totalP the total N and P content, and soilK, soilCa and soilMg the respective K, Ca and Mg concentrations).

	PC1	PC2
eigenvalue	4.77	2.03
variance	47.73	20.27
WHC	**0.87**	0.14
Sand	-0.58	**0.67**
Clay	0.35	**-0.79**
pH	**0.64**	-0.37
SOM	**0.84**	0.45
totalN	**0.79**	0.51
totalP	**0.83**	0.41
soilK	0.50	-0.34
soilCa	**0.80**	-0.23
soilMg	0.50	0.03

Table S10: Partial Kendall correlation coefficients between the plot average trait value and the four axes of environmental variation across the MEDIT network. Bold values indicate statistically significant associations (*p*<0.05), after controlling for the effect of all other environmental dimensions, and italic indicate marginally significant associations (*p*<0.1).

	T_{min}	P_{dq}	Soil Nutrient Status	Soil Texture
L_a	0.124	0.131	-0.161	-0.110
LMA	-0.008	**-0.230**	0.023	0.067
L_t	-0.036	-0.146	0.106	0.091
LDMC	0.107	**-0.336**	-0.066	0.077
C_m	-0.012	**-0.301**	0.043	0.168
N_m	0.032	**0.234**	-0.155	-0.031
P_m	**-0.182**	0.138	**-0.205**	-0.025
Ca_m	0.101	0.153	0.116	-0.061
Mg_m	0.083	0.069	-0.014	0.127
K_m	-0.105	**-0.232**	0.009	**-0.257**
$A_{sat,a}$	0.094	0.110	-0.146	0.019
$R_{dark,a}$	0.003	-0.158	0.086	-0.030
ρ_W	**0.265**	0.015	0.159	-0.124

Table S11: Optimal linear mixed effects models fitted for each measured trait, across the axes of environmental variation identified along the MEDIT forest plot network. In the fixed effects part bold values indicate statistically significant (p<0.05) and italics indicate marginally significant (p<0.1) coefficient estimates. In the random effects part, σ^2 represents the within group variance, τ_{Plot} and $\tau_{Species}$ the between group variances for species and plots respectively. τ11 indicates the environmental variable across which the slope of the trait – environmental relationship varies between species and the respective variance. ICC is the intraclass correlation coefficient and N_{plot}, $N_{species}$ and N the number of plots, species and total observations for each trait.

	L_a	*LMA*	L_t	*LDMC*	C_m	N_m	P_m	Ca_m	Mg_m	K_m	$A_{sat,a}$	$R_{dark,a}$	ρ_W
**Fixed Effects**													
intercept	-0.43	0.48	-0.25	**1.18**	**0.82**	-0.12	0.16	**-0.44**	-0.14	-0.02	-0.15	**0.78**	-0.28
LAI	**0.03**	**-0.05**										**-0.12**	
Tmin	-0.13						*-0.08*			**-0.08**			
Pdq			**0.01**	**-0.02**	**-0.01**		-0.01						
Soil Nutrient Status			-0.09	-0.08				0.06	**0.15**	**-0.14**			
Soil Texture	**0.05**	-0.08						0.03	**0.16**				
**Random Effects**													
σ^2	0.09	0.12	0.17	0.44	0.20	0.11	0.22	0.26	0.24	0.31	0.49	0.59	0.17
τ_{Plot}	0.00	0.03	0.13	0.40	0.09	0.10	0.16	0.10	0.09	0.11	0.16	0.28	0.07
τ_{pecies}	0.35	1.22	1.48	0.01	0.40	0.98	0.61	0.12	0.25	0.15	0.11	0.00	0.30
τ11	Tmin	Soil Texture	Soil Texture	Soil Nutrient Status				Soil Texture	Soil Nutrient Status				
	0.03	0.02	0.01	0.01				0.02	0.01				
ICC		0.91	0.91	0.51	0.70	0.91	0.78	0.48	0.62	0.45	0.36		0.68
N_{plot}	33	33	33	33	33	33	33	33	33	33	31	31	33
$N_{species}$	4	4	4	4	4	4	4	4	4	4	4	4	4
N	200	202	196	196	199	200	198	201	201	200	170	165	199

Figure S1: Geographic distribution of the MEDIT plot network. In total data from 61 plots were used. Study regions include some of the highest mountains in Greece. Starting from south to north: Mount Taygetos plot with TAY prefix, Mount Parnonas (PNAS), Mount Helmos (DOX), Mount Parnita (PTHA), Mount Dirfi (DIR), Mount Parnasos (PSOS), Mount Vardousia (VAR), Mount Oxia (GROX), Mount Agrafa (PLA), Valia Calda area (VLC), Mount Timfi (ZAG), Mount Kissavos (KIS), Mount Olympos (OLY), Mount Holomontas (HOL) and Mount Rodopi (ROD).

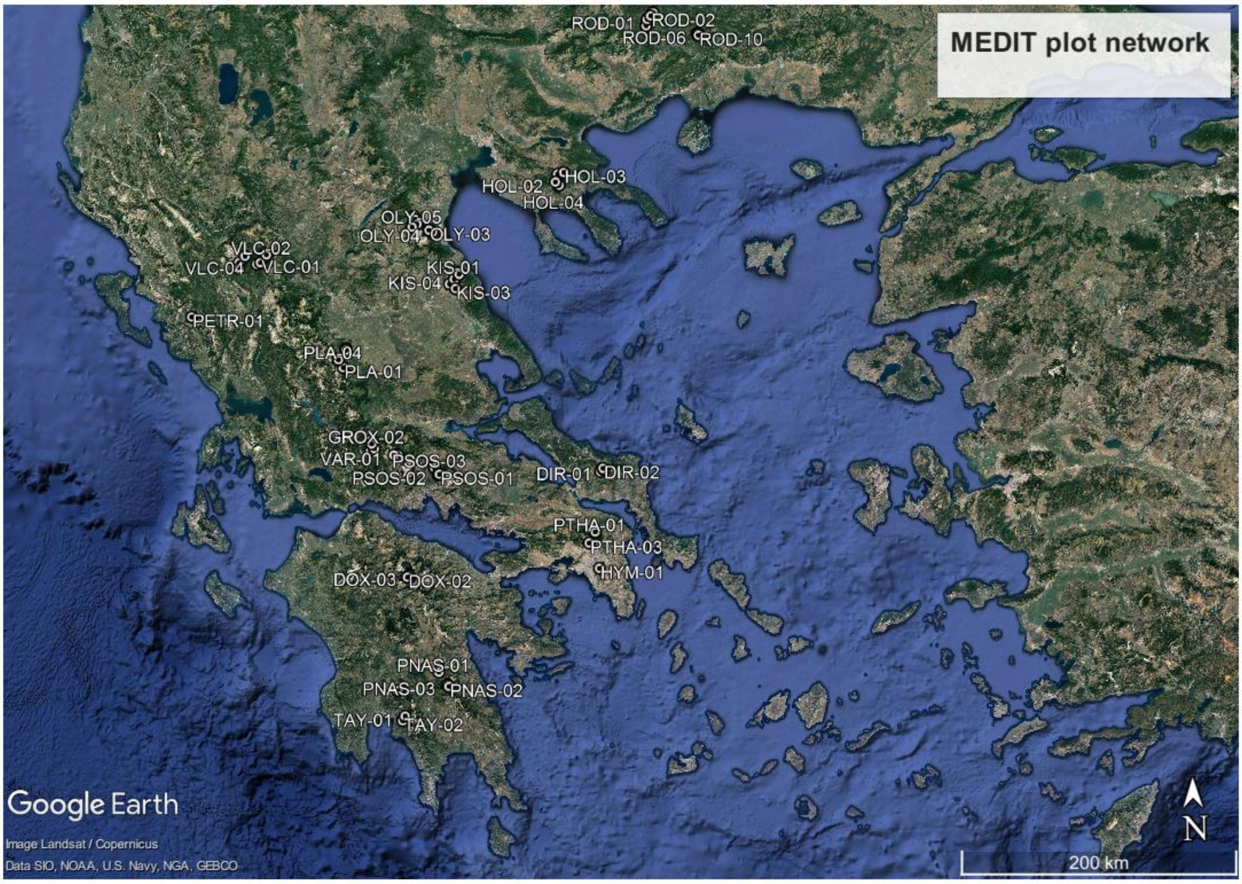

Figure S2: Trait-by-trait correlation analysis, across all PFTs and within needleleaf evergreen, broadleaf deciduous and broadleaf evergreen species. In all cases Pearson's correlation coefficients were estimated on the raw dataset, including intraspecific variation. The strength of the correlation is indicated by the colour bar. The cross indicates a non-significant correlation.

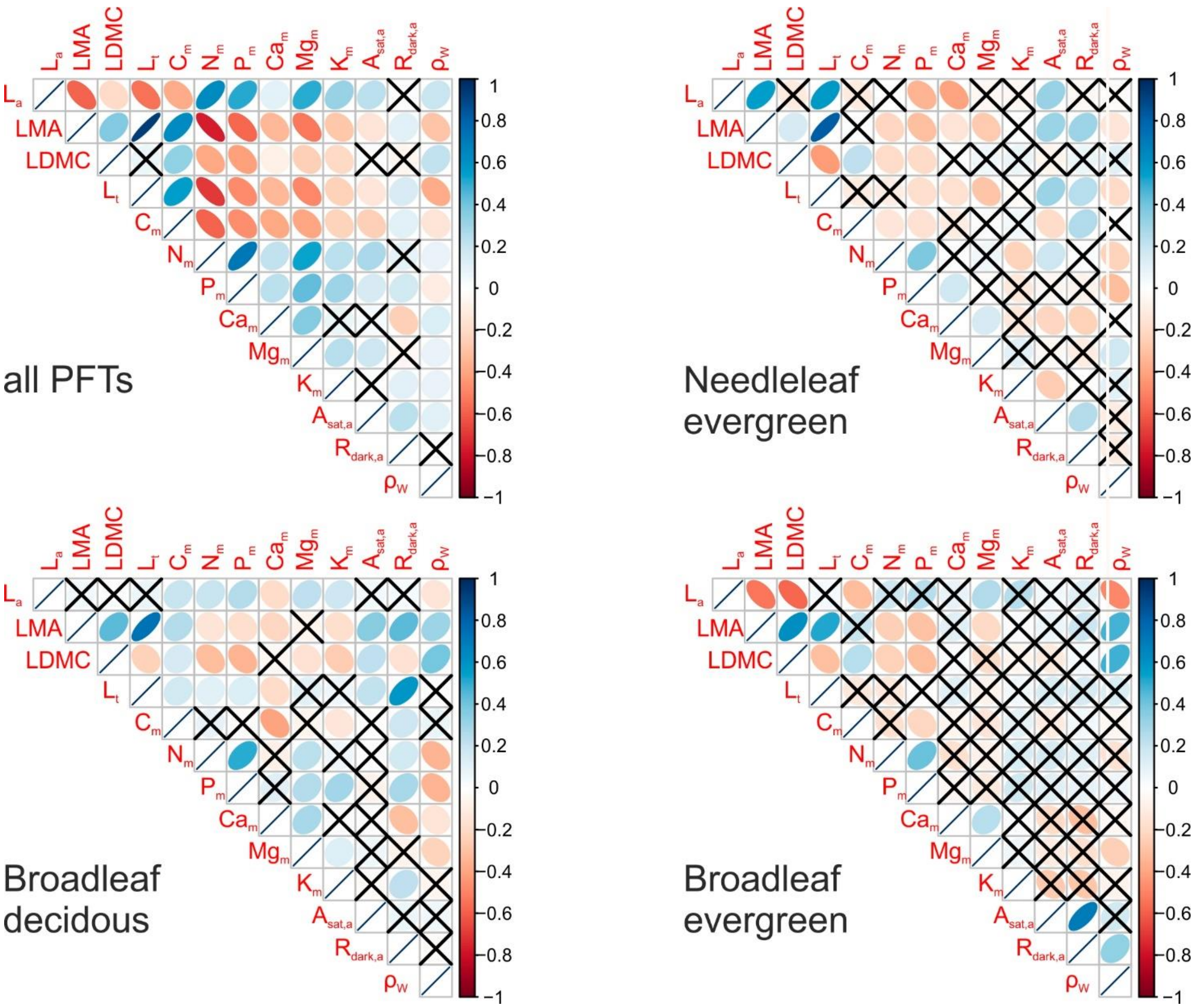

Figure S3: Significant relationships between the environmental component of trait variation and the four major environmental axes across the MEDIT plot network assessed with partial (T) Kendall correlation coefficients. The partial (T*) correlation coefficients of the raw plot level average trait value with the four environmental axes are also reported. Colours indicate plot dominance by different plant functional types (*Ne*: needleleaf evergreens - red, *Be*: broadleaf evergreens - blue, and *Bd* broadleaf deciduous - cyan).

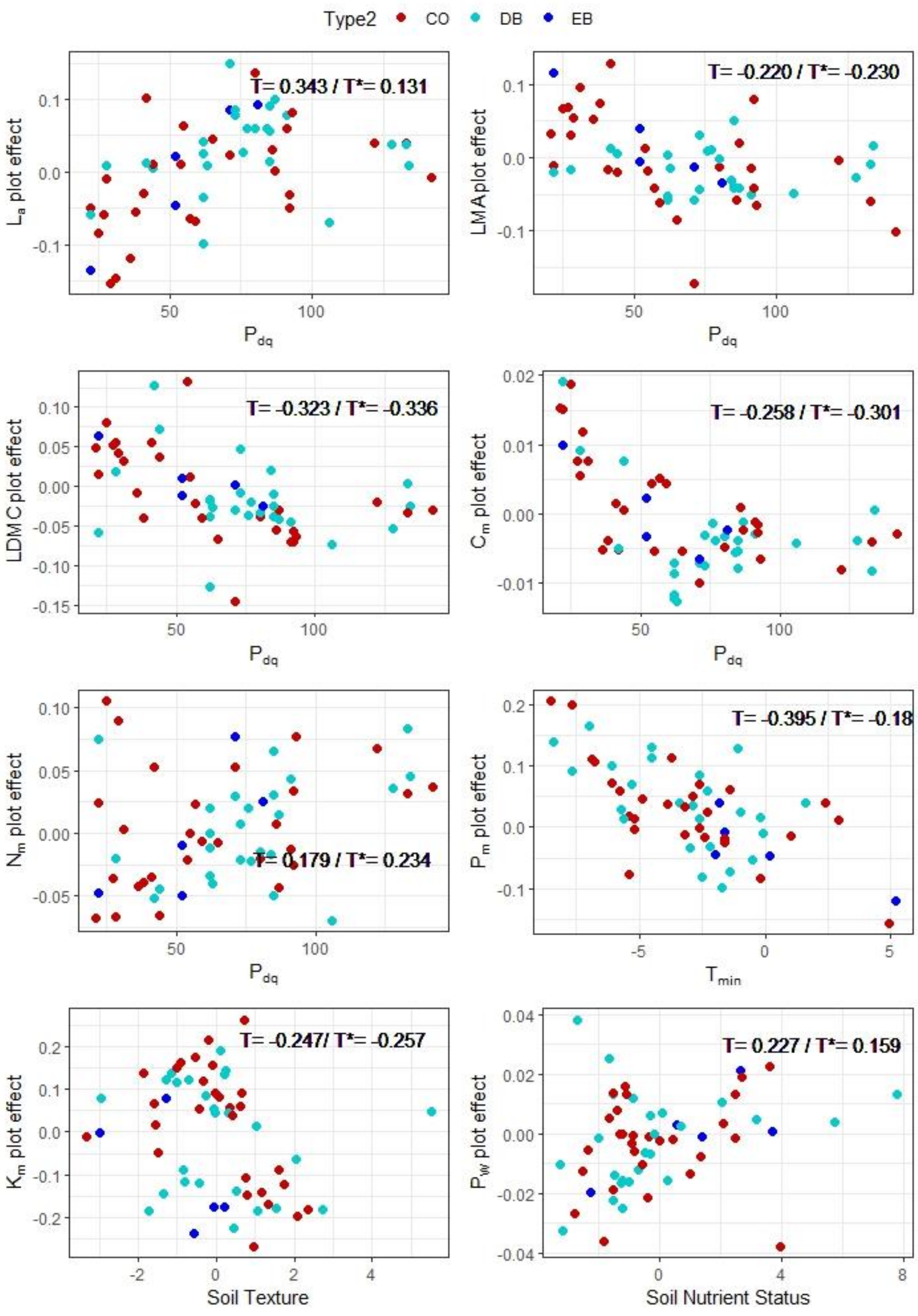

Supplement: Supplementary file 1 [file Data_Sheet_1.pdf]
